# Association of body composition in early pregnancy with gestational diabetes mellitus: A meta-analysis

**DOI:** 10.1371/journal.pone.0271068

**Published:** 2022-08-15

**Authors:** Fatemeh Alsadat Rahnemaei, Fatemeh Abdi, Reza Pakzad, Seyedeh Hajar Sharami, Fatemeh Mokhtari, Elham Kazemian

**Affiliations:** 1 Department of Obstetrics & Gynecology, Midwifery, Reproductive Health Research Center, Al-zahra Hospital, School of Medicine, Guilan University of Medical Sciences, Rasht, Iran; 2 Non-communicable Diseases Research Center, Alborz University of Medical Sciences, Karaj, Iran; 3 Epidemiology, Faculty of Health, Ilam University of Medical Sciences, Ilam, Iran; 4 Department of Obstetrics & Gynecology, Reproductive Health Research Center, Al-zahra Hospital, School of Medicine, Guilan University of Medical Sciences, Rasht, Iran; 5 Department of Midwifery, Reproductive Health, Faculty of Nursing and Midwifery, Isfahan University of Medical Sciences, Isfahan, Iran; 6 Department of Medicine, Samuel Oschin Comprehensive Cancer Institute, Cedars-Sinai Medical Center, Los Angeles, CA, Unites States of America; National Institute of Child Health and Human Development (NICHD), NIH, UNITED STATES

## Abstract

**Introduction:**

Body composition as dynamic indices constantly changes in pregnancy. The use of body composition indices in the early stages of pregnancy has recently been considered. Therefore, the current meta-analysis study was conducted to investigate the relationship between body composition in the early stages of pregnancy and gestational diabetes.

**Method:**

Valid databases searched for papers published from 2010 to December 2021 were based on PRISMA guideline. Newcastle Ottawa was used to assess the quality of the studies. For all analyses, STATA 14.0 was used. Mean difference (MD) of anthropometric indices was calculated between the GDM and Non-GDM groups. Pooled MD was estimated by “Metan” command, and heterogeneity was defined using Cochran’s *Q* test of heterogeneity, and *I* ^2^ index was used to quantify heterogeneity.

**Results:**

Finally, 29 studies with a sample size of 56438 met the criteria for entering the meta-analysis. Pooled MD of neck circumference, hip circumference, waist hip ratio, and visceral adipose tissue depth were, respectively, 1.00 cm (95% CI: 0.79 to 1.20) [N = 5; I^2: 0%; p: 0.709], 7.79 cm (95% CI: 2.27 to 13.31) [N = 5; I2: 84.3%; P<0.001], 0.03 (95% CI: 0.02 to 0.04) [N = 9; I2: 89.2%; P<0.001], and 7.74 cm (95% CI: 0.11 to 1.36) [N = 4; I^2: 95.8%; P<0.001].

**Conclusion:**

Increased neck circumference, waist circumference, hip circumference, arm circumference, waist to hip ratio, visceral fat depth, subcutaneous fat depth, and short stature increased the possibility of developing gestational diabetes. These indices can accurately, cost-effectively, and affordably assess the occurrence of gestational diabetes, thus preventing many consequences with early detection of gestational diabetes.

## Introduction

Gestational diabetes mellitus (GDM) is defined as carbohydrate intolerance with varying degrees that is first diagnosed in pregnancy [1]. GDM usually begins in the second half of pregnancy when the mother is unable to secrete enough insulin to compensate for the nutritional increase in pregnancy and the possible increase in fat and anti-insulin hormones that occur during pregnancy (such as human placental hormone, cortisol, and prolactin) [2]. GDM has many maternal and fetal consequences that can be both short-term and long-term [3].

Several risk factors increase GDM, including aging, GDM history, body mass index (BMI) greater than 30 kg/m^2^, family history of diabetes, history of a macrosomic infant weighing 4.5 kg, and race [4]. Other maternal complications include shoulder dystocia, preeclampsia, cesarean section, type-2 diabetes, metabolic syndrome, and cardiovascular disease [5–7]. Neonatal complications also include macrosomia, neonatal trauma, hypoglycemia, and other metabolic disorders of the neonatal period [8, 9].

Many maternal and neonatal complications can be improved by careful monitoring of blood glucose during pregnancy, medical treatments (insulin and metformin), diet, physical activity, and lifestyle changes [10, 11].

In 2010, the International Association of Diabetes and Pregnancy Study Groups (IADPSG) developed new diagnostic criteria for GDM, based for the first time on adverse pregnancy outcomes [12]. In 2013, the World Health Organization (WHO) defined the IADPSG criteria adjusted during the 75 g OGTT threshold to 1.75 times the odds ratio for adverse pregnancy outcomes by reducing fasting glucose concentrations by 5.1 ≥, 1-h ≥ 10, and/or 2-h ≥ 8.5 mmol per liter [13].

The global prevalence of gestational diabetes is estimated 1 to 28%; this difference is due to differences in the criteria for measuring GDM, age, race, ethnicity, lifestyle, and history of the populations in which the prevalence was measured [14–16].

Normal pregnancy is characterized by a physiological reduction of 50–60% in insulin sensitivity [17]. Studies have reported that the likelihood of GDM increases with maternal weight gain, especially in early pregnancy. Numerous studies have been conducted worldwide to identify effective risk predictors to support early prevention or treatment [18, 19].

Measurement of body composition seems to be a practical method for potential screening of GDM [20]. Body composition is a risk factor for conditions such as diabetes, preeclampsia, and gestational hypertension [21, 22]. Obesity is a powerful predictor of GDM, and abdominal obesity is a powerful factor in the development of GDM and future diabetes [23, 24]. However, obesity is a complex process in which the distribution of body fat is involved, and body fat leads to adverse metabolic and cardiovascular consequences [25]. Studies show that increasing body composition, especially body fat, is closely related to glucose metabolism in humans [26]. But data on body composition and anthropometric indices are low. Studies show that weight gain in the first 2–3 months is composed of more fat mass, and patients with higher BMI gain more fat mass [15, 16] which can affect subsequent maternal insulin resistance [27].

However, there are other anthropometric indices that have been considered recently. In addition to showing more accurate information about body composition, they can also predict pregnancy outcomes, including GDM in pregnant women. For example, measurement of visceral abdominal adipose tissue (VAT) [28], neck circumference (NC), hip circumference (HC) and waist circumference (WC) [29], percentage of skeletal muscle mass and percentage of fat mass [30], and central obesity [31] can be used as an approach to predict occurrence GDM. Previous meta-analysis studies have shown a direct relationship with indices of general body obesity including WC, waist to hip ratio (WHR), and VAT with GDM [32].

In this study, according to the time period searched (1985–2020), a small number of studies were analyzed; in addition, a small number of anthropometric indices indicating the body composition were examined. Therefore, the present study was performed by reviewing the updated studies and all anthropometric indices expressed in the studies and using an accurate model in the early stages of pregnancy in a systematic review and meta-analysis to investigate the relationship between anthropometric indices expressing body composition and GDM.

## Materials and methods

This study was approved by Alborz University of Medical Sciences (ethnical code: IR.ABZUMS.REC.1400.241). Preferred Reporting Items for Systematic Reviews and Meta-Analyses (PRISMA) guidelines were observed in the report of the study. PRISMA contains 27 items related to the content of a systematic and meta-analysis, and includes abstracts, methods, results, discussions, and financial resource [33, 34]. Participant consent for this study is not applicable. This study was registered on PROSPERO website by "CRD42022302813" ID.

### Search strategy

PubMed, Web of Science, Scopus, Google Scholar, and ProQuest were searched from 2010 to December 2021. MESH keywords and search strategy were as below:

1. ’Gestational diabetes’ [tiab], OR ’GD’ [tiab], OR ’Gestational Diabetes Mellitus’ [tiab], OR ’GDM’ [tiab], OR ’pregnancy induced diabetes’[tiab]
2. ’Anthropometric indicators’ [tiab], ’Anthropometric indices’ [tiab], OR ’body size’[tiab], OR ’body composition’ [tiab] OR, ’Waist/Hip Ratio’ [tiab], OR ’WHR’ [tiab], OR ’ visceral fat mass’ [tiab], OR ’VFM’ [tiab], OR ’ Neck circumference’ [tiab], OR ’hip circumference’ [tiab], OR ’ waist circumference’ [tiab], OR ’ subcutaneous adipose tissue’ [tiab], OR ’ skeletal muscle mass percentage’ [tiab], ’total adipose tissue thickness’ [tiab], OR ’subcutaneous adipose tissue’[tiab], OR ’Subcutaneous fat thickness’ [tiab], OR ’visceral adipose tissue depth’ [tiab], OR ’skinfold thickness’ [tiab], OR ’mid upper arm circumference’ [tiab], OR ’subcutaneous fat thickness’ [tiab], OR ’fat mass percentage’ [tiab], OR ’fat mass index’ [tiab], OR ’muscle mass percentage’ [tiab], OR ’Skinfold Thickness’ [tiab]
3. ’Pregnancy’ [tiab], OR ’Pregnancies’ [tiab], OR ’Gestation’[tiab], OR ’early pregnancy’ [tiab]
4. #1 AND #2
5. #1 AND #2 AND #3

### Eligibility criteria

#### Inclusion and exclusion criteria

We set our inclusion and exclusion criteria based on PICO criteria (population, intervention, comparison, outcome, and study design) (Table 1).

**Table 1:** PICO criteria.

Selection criteria	Inclusion criteria	Exclusion criteria
**Population**	Healthy pregnant women with single fetus and at reproductive age group, GDM based on the diagnostic criteria, Gestational age considered for each study based on ultrasound, Studies were published until December 2021, Full-text available and with no language restrictions	Multiple pregnancies, women taking steroids, pre-pregnancy diabetes, maternal medical disorders such as liver, kidney, thyroid, fetal abnormalities, ovarian cysts, and maternal age less than 18 years
**Exposure**	Body composition (WHR, visceral adipose mass, NC, HCWC, subcutaneous adipose tissue (SAT), skeletal muscle mass percentage(SMMP), total adipose tissue thickness(TAT), VAT, skinfold thickness, mid upper arm circumference(MUAC), fat mass percentage(FMP), fat mass index(FMI), muscle mass percentage(MMP), skinfold thickness	Other body composition
**Comparison**	Healthy control group	GDM was combined with other maternal pregnancy complications (HDP, eclampsia, and pre-eclampsia); ethnicity, food habits, and separation were difficult.
**Outcome**	GDM according to different screening protocols	-
**Study design**	Cohort, case control, and cross sectional	Case study, case series, case report, lack of access to full text articles, review articles, letter to editor

### Study selection

**Fig 1:**
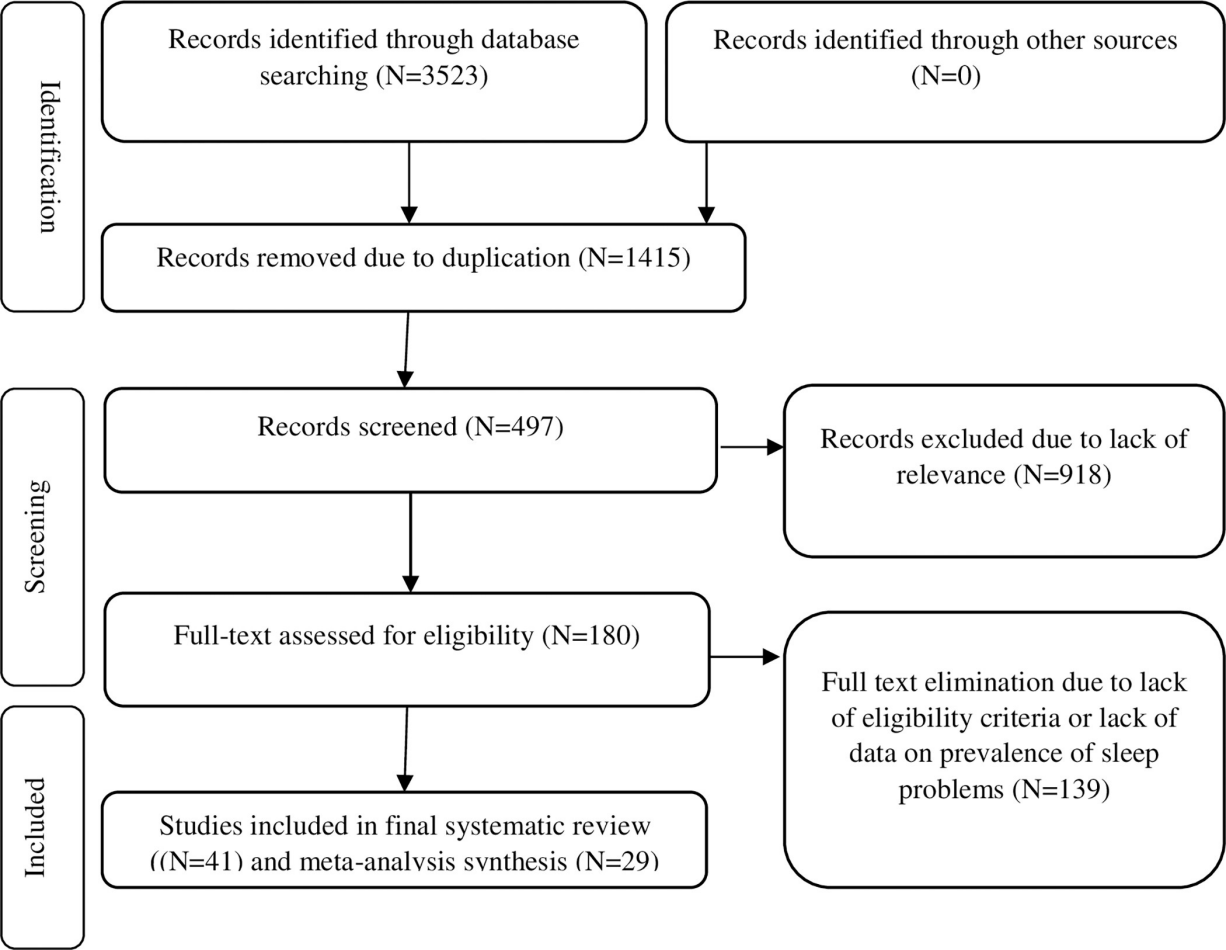
PRISMA flowchart of selected studies.

The initial search yielded 3523 results. The eligibility of these articles was independently evaluated by two authors, and disagreements were resolved by consensus. In the first stage, 2108 irrelevant or duplicate articles were excluded. After reviewing the titles and abstracts of the remaining articles, 918 more papers were excluded. In the evaluation of the full texts, 139 ineligible articles were excluded out of the remaining 180 articles. Finally, a total of 41 eligible articles were reviewed and 29 articles meets criteria to meta-analysis (Fig 1).

### Quality assessment

Newcastle Ottawa scale (NOS) was used to measure the quality of studies. This scale is used to measure the quality of cohort and case control studies. The validity and reliability of this tool have been proven in various studies [35, 36].

### Data extraction

Two authors independently performed the study selection and validity assessment and resolved any disagreements by consulting a third researcher. Author, year, study design, geographic region, maternal age, diagnostic criteria of GDM, anthropometric indices, accompanying factors, results, and quality assessment scores were extracted from articles.

### Statistical analysis

All analyses were conducted with STATA 14.0 (College Station, Texas). For each study, mean value and standard deviation (SD) of anthropometric indices were extracted; if IQR was reported, we changed it to SD with IQR/1.35. Then, the mean difference (MD) of anthropometric indices was calculated between GDM and non-GDM group for each study. Then, standard error (SE) of MD was calculated for each study using the following formula:

$${\mathrm{SE}}_{\mathrm{MD}}=\sqrt{\frac{\sigma_{1}^{2}}{n_{1}}+\frac{\sigma_{2}^{2}}{n_{2}}}$$

Where, $\sigma_{1}^{2}$, n_1,_ $\sigma_{1}^{2}$, and n_2_ are variance values, and samples size in GDM and control groups, respectively. Then, pooled MD was calculated by “Metan” command [37]. Heterogeneity was determined using Cochran’s *Q* test of heterogeneity, and the *I* ^2^ index was used to quantify heterogeneity. In accordance with Higgins classification approach, *I* ^2^ values above 0.7 were considered as having high heterogeneity. To estimate the pooled MD for anthropometric indices, the fixed-effect model was used; when heterogeneity was greater than 0.7, the random effects model was used. The meta-regression analysis was used to examine the effect of publication year, age, sample size, and study design as factors affecting heterogeneity among studies. The “meta bias” command [38] was used to check for publication bias, and if there was any publication bias, the pooled MD was adjusted with the “meta trim” command using the trim-and-fill method [39]. In all analyses, significance level was considered 0.05.

## Results

Twenty-nine studies with a sample size of 56,438 met the meta-analysis inclusion criteria (Table 1). Fig 1 shows the flowchart of the study selection process. Anthropometric indices values for the groups with and without GDM of included studies are given in Table 5.

### Pooled MD of anthropometric indices

**Fig 2:**
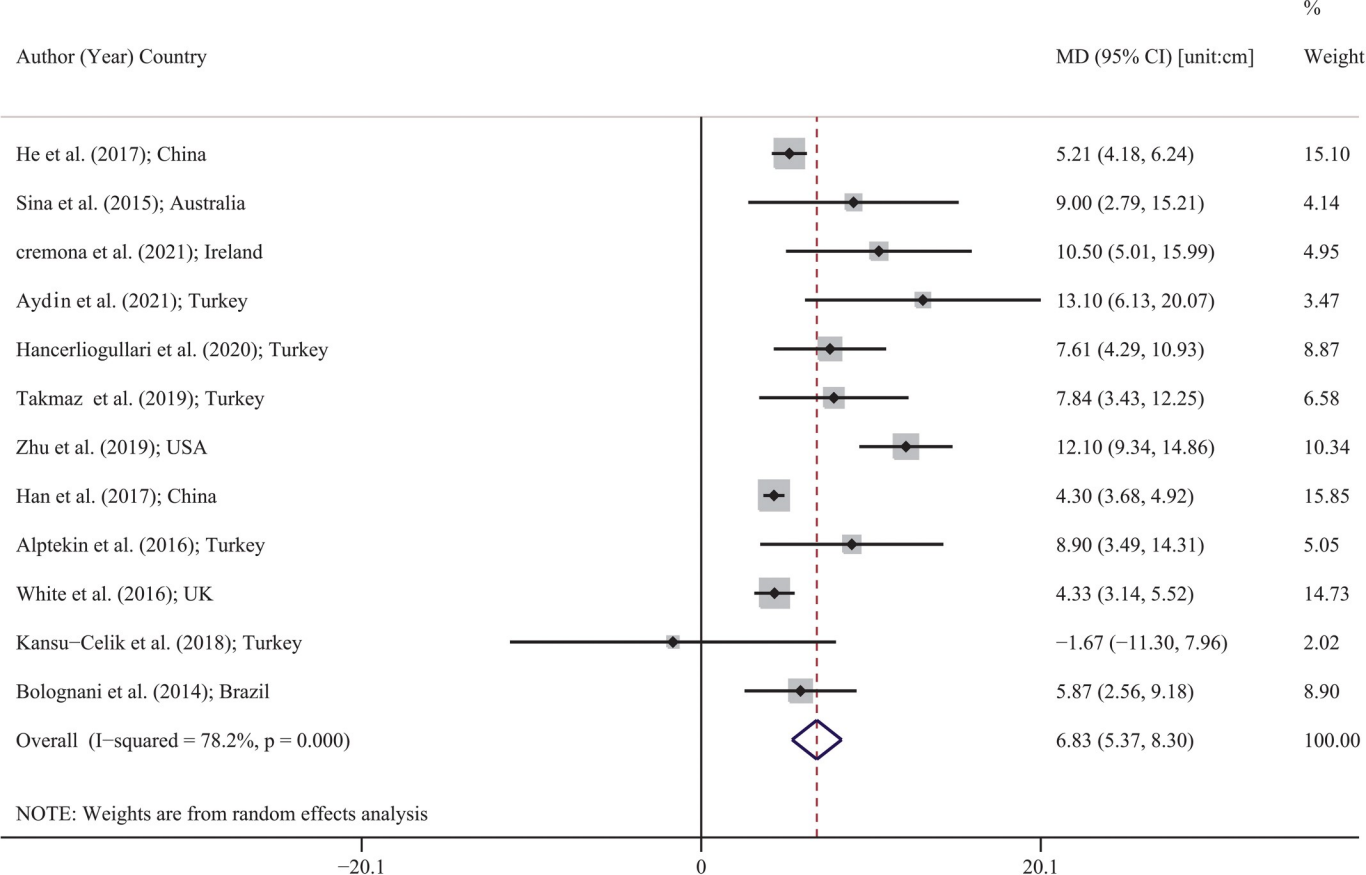
Forest plot for MD of waist circumference (cm) between GMD and non-GDM group based on a random effects model. Each study is distinguished by its author (year) and countries. Each line segment’s midpoint shows the MD estimate; the length of line segment indicates 95% confidence interval (CI) in each study, and the diamond mark illustrates the pooled estimate of MD.

**Fig 3:**
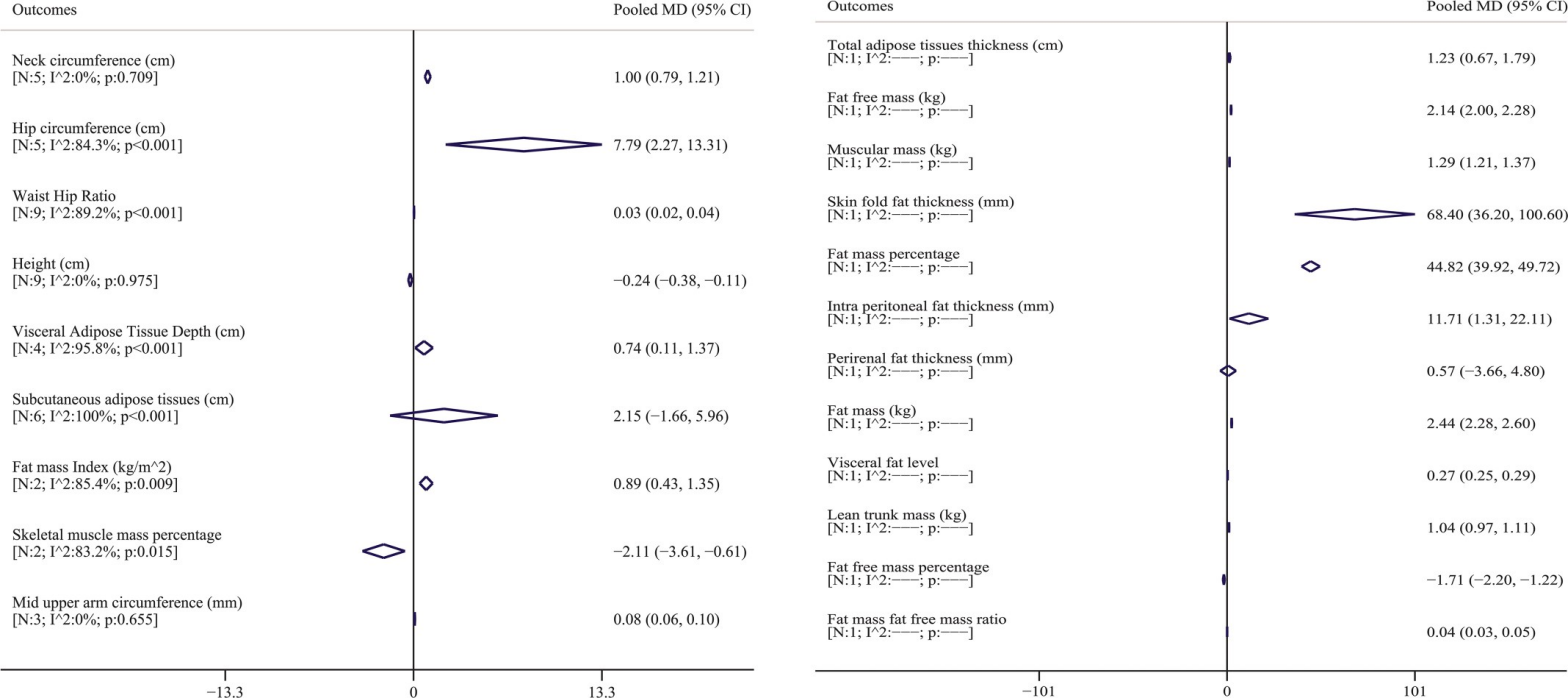
Pooled MD and 95% confidence interval of anthropometric index. The diamond mark illustrates the pooled MD, and the length of the diamond indicates 95% CI.

Table 2 shows the pooled MD of all anthropometric indices. As shown in Table 2, twelve studies were carried out for waist circumference, five studies for neck and hip circumference, nine studies for waist hip ratio and height, six studies for subcutaneous adipose tissues, four studies for visceral adipose tissue depth, three studies for mid upper arm circumference, two studies for fat mass index and skeletal muscle mass percentage, and one study for other indices. Fig 2 shows the pooled MD of waist circumference for included studies. The lowest and highest MDs were reported by Kansu-Celik et al. [40] in Turkey (MD: -1.67; 95% CI: -11.30 to 7.96) and Aydin et al. [41] in Turkey (MD: 13.10; 95% CI: 6.13 to 20.07). Based on random effects model, the pooled MD for waist circumference was 6.83 cm (95% CI: 5.37 to 8.30). In other words, the mean values of waist circumference in people with GDM were higher than that in non-GDM people. Forest plot of other anthropometric indices was provided in supplements 1 to 21, and pooled MD is shown in Table 2 and Fig 3. Pooled MD of neck circumference, hip circumference, waist hip ratio, and visceral adipose tissue depth was 1.00 cm (95% CI: 0.79 to 1.20) [N = 5; I^2: 0%; p: 0.709]; 7.79 cm (95% CI: 2.27 to 13.31) [N = 5; I^2: 84.3%; P<0.001]; 0.03 (95% CI: 0.02 to 0.04) [N = 9; I^2: 89.2%; p<0.001] and 7.74 cm (95% CI: 0.11 to 1.36) [N = 4; I^2: 95.8%; P<0.001], respectively, which indicates that the average of these indices was higher in the GDM group. An adverse pattern was observed for the height and skeletal muscle mass percentage, which pooled MD for the height, and skeletal muscle mass percentage was -0.24 cm (95% CI: -0.37 to -0.10) [N = 9; I^2: 0%; p:0.975]; and -2.11 (95% CI: -3.61 to -0.61) [N = 2; I^2: 83.2%; p:0.015], respectively, which indicates that the average of these indices was higher in the non-GDM group. In other words, in general, people with non-GDM had a mean height and skeletal muscle mass percentage higher than GDM people. Although pooled MD was higher for subcutaneous adipose tissues in the GDM group, this difference was not significant (2.15 [95% CI: -1.66 to 5.96]). The pooled MD of other indices are given in Table 2 and Fig 3.

**Table 2:** Pooled MD (95% confidence interval) and heterogeneity of anthropometric indices.

Outcomes	Heterogeneity index	Number of studies	Pooled MD (95% CI) ^#^
Waist circumference (cm)	I^2: 78.2%; p<0.001	12	6.83 (5.37 to 8.30) *
Neck circumference (cm)	I^2: 0%; p: 0.709	5	1.00 (0.79 to 1.20) *
Hip circumference (cm)	I^2: 84.3%; p<0.001	5	7.79 (2.27 to 13.31) *
Waist Hip Ratio	I^2: 89.2%; p<0.001	9	0.03 (0.02 to 0.04) *
Height (cm)	I^2: 0%; p: 0.975	9	-0.24 (-0.37 to -0.10) *
Visceral Adipose Tissue Depth (cm)	I^2: 95.8%; p<0.001	4	0.74 (0.11 to 1.36) *
Fat mass percentage	I^2: ---; p: ---	1	44.82 (39.92 to 49.72) *
Subcutaneous adipose tissues (cm)	I^2: 100%; p<0.001	6	2.15 (-1.66 to 5.96)
Total adipose tissues thickness (cm)	I^2: ---; p: ---	1	1.23 (0.67 to 1.79) *
Fat mass Index (kg/m^2)	I^2: 85.4%; p: 0.009	2	0.89 (0.43 to 1.35) *
Skeletal muscle mass percentage	I^2: 83.2%; p: 0.015	2	-2.11 (-3.61 to -0.61) *
Fat free mass (42)	I^2: ---; p: ---	1	2.14 (2.00 to 2.28) *
Muscular mass [42]	I^2: ---; p: ---	1	1.29 (1.21 to 1.37) *
Skin fold fat thickness (mm)	I^2: ---; p: ---	1	68.40 (36.20 to 100.6) *
Mid upper arm circumference (mm)	I^2: 0%; p: 0.655	3	0.08 (0.06 to 0.10) *
Intra peritoneal fat thickness (mm)	I^2: ---; p: ---	1	11.71 (1.31 to 22.11) *
Perirenal fat thickness (mm)	I^2: ---; p: ---	1	0.57 (-3.66 to 4.80)
Fat mass [42]	I^2: ---; p: ---	1	2.44 (2.28 to 2.60) *
Visceral fat level	I^2: ---; p: ---	1	0.27 (0.25 to 0.29) *
Lean trunk mass [42]	I^2: ---; p: ---	1	1.04 (0.97 to 1.11) *
Fat free mass percentage	I^2: ---; p: ---	1	-1.71 (-2.20 to -1.22) *
Fat mass fat free mass ratio	I^2: ---; p: ---	1	0.04 (0.03 to 0.05) *

CI: Confidence Interval

*: significant

# Positive pooled MD means the index was higher in GDM compared to non-GDM, and negative pooled MD means the index was lower in GDM compared to non-GDM.

### Heterogeneity and meta-regression results

**Fig 4:**
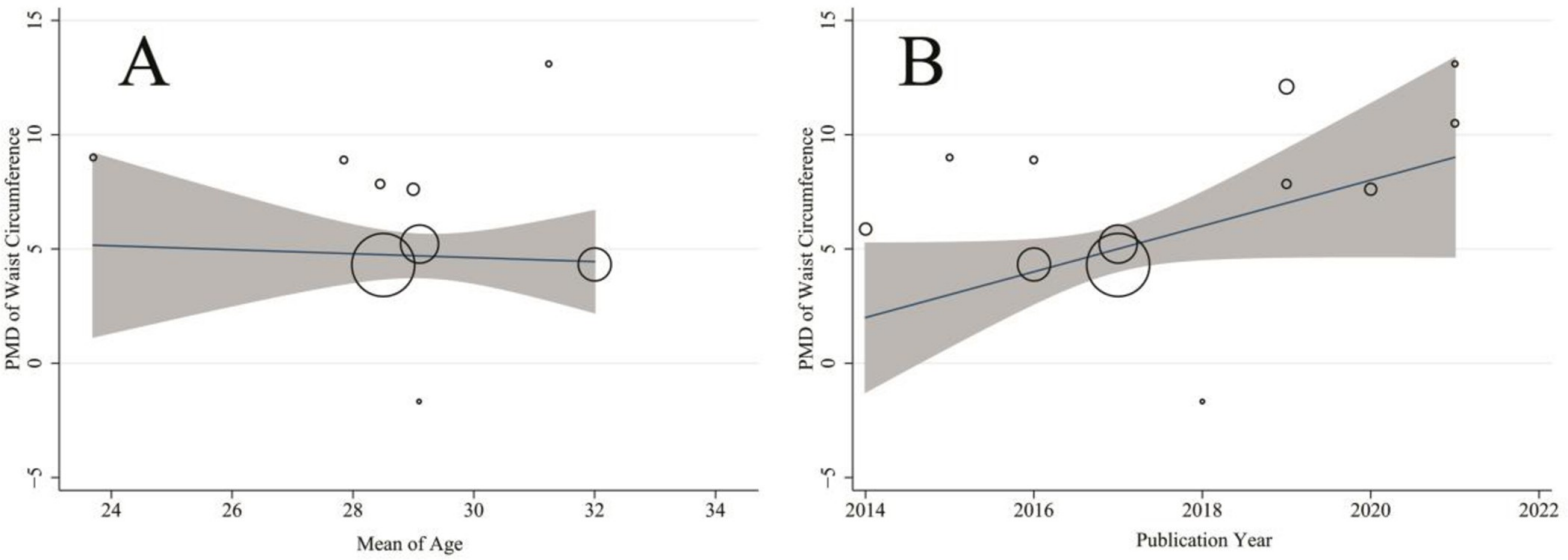
Association between pooled mean difference (MD) of waist circumference with age (A) and publication year (B) by means of meta regression. The size of circles indicates the precision of each study. There is no significant association with respect to the pooled MD of waist circumference with age publication year.

Table 2 shows significant heterogeneity between different studies for waist circumference, hip circumference, waist/hip ratio, visceral adipose tissue depth, subcutaneous adipose tissue (Cochran’s Q test P-value < 0.001 for all lipid profiles) so that the I^2^ index was above 70% for all mentioned indices. Table 3 shows the meta-regression results to investigate the effect of publication year, age, sample size, and study design on heterogeneity between studies. Accordingly, none of the variables had a significant role on heterogeneity between studies (P>0.05 for all). Fig 4 shows the result of meta-regression for association between pooled MD of waist circumference with age (A) and publication year (B).

**Table 3:** Results of the univariate meta-regression analysis on the heterogeneity of the determinant.

variables	Publication Year (year)		Age		Sample size		Study Design*	
	Coefficient 95% CI	p-value	Coefficient 95% CI	P-value	Coefficient 95% CI	P-value	Coefficient 95% CI	P-value
Waist Circumference	0.81 (-0.11 to 1.75)	0.078	-0.36 (-1.48 to 0.77)	0.480	0.01 (-0.01 to 0.01)	0.656	-0.88 (-4.98 to 3.23)	0.643
Hip Circumference	1.60 (-0.66 to 3.86)	0.109	-0.29 (-3.62 to 3.04)	0.743	0.01 (-0.02 to 0.01)	0.071	0.94 (-21.17 to 23.06)	0.900
Waist/Hip Ratio	-0.01 (-0.01 to 0.01)	0.979	-0.01 (-0.01 to 0.01)	0.067	0.01 (-0.01 to 0.01)	0.705	0.01 (-0.01 to 0.03)	0.280
Visceral Adipose Tissue Depth	0.22 (-0.43 to 0.88)	0.276	-0.13 (-0.34 to 0.08)	0.081	0.01 (-0.01 to 0.01)	0.633	0.92 (-0.57 to 2.41)	0.116
Subcutaneous adipose tissue	-1.02 (-3.48 to 1.44)	0.313	1.59 (-0.89 to 4.08)	0.134	-0.01 (-0.07 to 0.06)	0.846	-2.69 (-8.49 to 3.12)	0.268

CI: Confidence Interval

*: Significant

Coding for study design: 1 = case control; 2 = cohort; 3 = cross-sectional

Table 4 shows the publication bias results based on the Egger’s test and the fill and trim method. There was a significant publication bias for waist circumference (coefficient: 1.95; P: 0.019) and hip circumference (coefficient: 3.06; P: 0.028). According to the fill and trim method, the value of adjusted pooled MD for waist circumference and hip circumference was 5.35 (95% CI: 3.81–6.88) and 7.80 (95% CI: 2.76–13.31), which was not significantly different from the pooled MD calculated for waist circumference (6.83 [95% CI: 5.37–8.30]) and hip circumference (7.79 [95% CI: 2.27–13.31]). In other words, the publication bias had no cosiderable effect on the result of meta analysis. No publication bias was observed for other anthropometric indices including neck circumference, waist/hip ratio, height, visceral adipose tissue depth, and subcutaneous adipose tissue. Details of the studies are listed in Table 5.

**Table 4:** Result of publication bias for anthropometric indices and fill and trim method result of adjusting publication bias.

Variables	Publication bias		Trim and fill	
	Coefficient 95% CI	p-value	Coefficient 95% CI	p-value
Waist Circumference	1.95 (2.57 to 5.09)	0.019*	5.35 (3.81 to 6.88)	<0.001
Neck Circumference	0.26 (-2.59 to 3.12)	0.788	---	
Hip Circumference	3.06 (0.64 to 5.49)	0.028*	7.80 (2.76 to 13.31)	<0.001
Waist/Hip Ratio	2.83 (-0.48 to 6.15)	0.083	---	
Height	0.11 (-0.39 to 0.62)	0.608	---	
Visceral Adipose Tissue Depth	6.75 (-0.41 to 13.91)	0.056	---	
Subcutaneous adipose tissue	-1.94 (-136.42 to 132.55)	0.970	---	

CI: Confidence Interval

*: Significant

**Table 5:** Details of studies included in the systematic review.

ID	References	Study design	Sample size	Geographic region	Age(year)	Diagnostic criteria of GDM	Anthropometric indices	applying Time	Accompanying factors	Results	QS
1	Jitngamsujarit et al.(2021) [43]	Cross-sectional	212	Thailand	27.1 ± 6.7	WHO	WC≥82: (OR 7.85, 95%CI 1.80–34.32	<18	• maternal age • history of diabetes in family • history of giving birth to a fetal anomaly • History of giving birth to an infant ≥ 4,000 gm	Significant	8
2	Saif Elnasr et al.2021 [44]	Cohort	83	Egypt	26.8	ADA	VAT: 5.85 ± 0.47 cm SAT:1.80±0.57 cm	11–14	BMI	VAT depth ranged from 1.4 to 9.1 cm, with a mean of 3.9 ± 1.6 cm is associated with GDM.	8
3	Cremona et al.2021 [45]	Cohort	187	Ireland	18–50	IADPSG	• abdominal SAT:1.99 (1.64–2.31) mm • abdominal VAT:1.41 (1.11–1.65) mm • FMP: 45.6 (39.2–49.0) • MUAC:32.9 (30.1–36.4) cm • WC = 90.3 (85.9–96.2) cm • HC: 108.6 (99.9–111.6) cm total SFT:226.4 (184.1–244.7) mm	10–16	• BMI • Parity >3 • Family Hx diabetes Age >40 • Smoking • High risk ethnicity • Previous perinatal death • Glucosuria • Previous baby ≥4.0 kg • Previous macrosomia (≥4.5 kg)	Significant for VAT, SAT, WC, HC and total SFT	7
4	Barforoush et al.2021 [46]	cohort	372	Iran	28.1 ±4.4	ADA	NC: 35.1 ±2.7 cm	14–16	Age Gravidity Family -history of type 2 diabetes Pre-pregnancy weight Height	NC ≥34.3 cm can be deemed as a predictor of GDM	8
5	Aydin et al.2021 [41]	Cohort	142	Turkey	31.24±5.11	IADPSG	• Intraperitoneal fat thickness:51.59 ± 22.49 mm • SAT: 19.79 ± 12.52 mm • WC:95.25±15 cm HC:115.38±15.41 cm WHR: 0.82±0.06 cm Perirenal fat thickness: 11.77±8.79 mm, SFT_max_: 19.79±12.52 mm	11–14	• Pre-pregnancy BMI • BMI • smoking • history of DM in the first degree relatives • GDM during previous pregnancy	Significant for all except Perirenal fat thickness	7
6	Zhang et al.2020 [47]	Cohort	22,223	China	28.09 ± 4.48	IADPSG	FM: 17.95 ± 5.65 kg, 1.085 (1.079–1.091) FFM: 40.56 ± 4.92 kg, 1.080 (1.100–1.115) Fat mass percentage: 30.09 ± 5.69%, 1.057 (1.052–1.063) MM:21.87 ± 2.96 kg, 1.114 (1.106–1.121) VF level:8.48 ± 0.56, 2.604 (2.459–2.758) Lean trunk mass: 18.32 ± 2.47 kg, 1.226 (1.209–1.243)	<17	• BMI • Total body water • Proteins • Bone minerals • Basal metabolic rate	Significant	7
7	Rocha et al.2020 [48]	Cohort	133	Brazil	26±6.2	IADSPG	VAT: 55.4 ±11.4 mm	≤20	BMI	Significant	9
8	Alves et al.2020 [28]	cohort	518	Brazil	26.25±5.8	IADPSG	VAT: 5.44 ±1.27mm	14	• age • Pre-pregnancy BMI	significant	8
9	Hancerliogullari et al.2020 [29]	cohort	525	Turkey	27 (18–44)	Carpenter and Coustan	NC:37.14 ± 3.34 cm WC: 91.78 ± 11.41 cm	11–14	• Age • Parity • BMI	Significant	8
10	Liu et al.2020 [30]	cohort	1318	China	32.6±5.1	IADPSG	FMI: 7.14±2.26 SMMP: 40.0±8.3 FMP: 30.1±5.8	13	• Age • pre-pregnancy BMI • Pre-pregnancy weight	Significant	8
11	Thaware et al.2019 [49]	Cohort	80	UK	18–40	IADPSG /WHO	VAT: 4.36±1.31 cm SAT: 2.24±1.01 cm	9–18	• Early pregnancy BMI ≥30 kg/m2 • Family history of diabetes in first-degree relative	Significant for VAT of ≥ 4.27 cm (p = 0.03)	8
12	Takmaz et al.2019 [50]	cohort	261	Turkey	30.57±5.78	IADPSG	WC: 103.91±14.13 cm 8.36(0.74–0.84)	20–24	• Age • Parity • Weight gain • PPBMI • BMI	Significant	7
13	Budak et al.2019 [42]	Case control	100	Turkey	33.5 (27–37)	Carpenter and Coustan	SFT: 21.1 (16.6–26.4)**mm	24–28	• Age • Parity • Weight gain	Significant	9
14	Kawanabe et al.2019 [51]	Cohort	96	Japan	34.4 ± 4.8	IADPSG	ASM: 17.0 ± 2.1 kg FM: 18.8 ± 8.2 kg ASM/FM ratio: 1.02 ± 0.34	16–30	• ISI • Age • HbA1c • pre-pregnancy BMI • Family history of diabetes	Significant	8
15	Marshall et al.2019 [52]	cohort	1,775,984	California	18–40	ICD-9	MH: 1.68 (1.58–1.66) m	nine months prior to birth	• Age • BMI	Taller women were less likely to have GDM 0.81 (0.80, 0.82)*.	8
16	Ulubasoglu et al.2019 [53]	cohort	148	Turkey	28.4±3.8	ADA	WC = 87.7 ±13.6 cm	11–14	• Total triglycerides • BMI	Significant	8
17	Wang et al.2019 [54]	Case-control	2698	China	30.95± 4.01	IADPSG	• FFMP: 68.45±4.81 • FMP: 31.55±4.81 FMI: 7.00±1.81 WHR: 0.86±0.04 MUAC: 27.64±2.30 cm FM/FFM ratio: 0.47 ±0.14	13–20	• Age • PPBMI	Significant	7
18	Zhu et al.2019 [31]	Cohort	1750	California	18–45	Carpenter and Coustan	WHR = 0.91 ±0.06 WC = 102.4 ±18.5 cm	10–13	• Smoking • Family history of diabetes • Previous GDM • Preexisting hypertension • Physical inactivity in early pregnancy	Significant	7
19	Nombo et al.2018 [55]	Cross sectional	609	Tanzania	27.5 ± 5.0	WHO	MUAC = 27.3± 3.8 cm	20–38	• Previous stillbirth • Family history of type 2 diabetes • Diet habits	Significant	9
20	Anafcheh et al.2018 [56]	Case control	195	Iran	32.35± 0.68	WHO	H = 159.72±6.72	<24–28	• Blood group • GWG • Age • History of stillbirth • History of GDM • History of type 2 diabetes in first-degree relatives • Birth -History of a baby weighing≥ 4 kg • History of a birth with a congenital anomaly • History of PCO	NS	7
21	Balani et al.2018 [57]	cohort	302	UK	31	WHO	PBF VFM<210 WHR	15	Age BMI • History of PCOs • Family history of diabetes, • History of hypertension and Previous macrosomia	Significant	7
22	Bourdages et al.2018 [58]	cohort	1048	Canada	28.9 ± 4.1	IADPSG	• SAT: 0.66 (0.59–0.73) • TAT:0.68 (0.61–0.76) • VAT: 0.65 (0.58–0.73)***	11–14	• Age≥35 • BMI≥31.6	Significant	8
23	Kansu-Celik et al.2018 [40]	Cross sectional	223	Turkey	27.46± 5.9	Carpenter and Coustan	• SAT: 19 (11–28) mm • WC: 95 (72–111) cm • WHR: 0.89 ± 0.59	24–28	• BMI	Significant	9
24	KhushBakht et al. 2018 [59]	Cross sectional	90	Pakistan	30.8 ± 3.2	ADA	• NC: 36.1 ± 2.8 cm • H: 1.61 ± 0.03 m • WC: 104.2 ± 9.0 cm	16	• BMI • Fasting lipid profile • Serum albumin • Uric acid • Age Gravidity	cut-off value of neck circumference for predicting GDM was 35.70 cm with a sensitivity of 51.4% and specificity of 81.2%.	9
25	Nassr et al.2018 [60]	cohort	389	USA	29.7±4.67	ACOG	Pre-peritoneal fat: 12 (9–16)**** mm SFT: 11 (8–14) mm BFI: 0.78 (0.42 - 1.26)	18–24	• Age>30 • Parity • History of GDM • History of bariatric surgery • Current gestational hypertension or preeclampsia	Significant	8
26	D’Ambrosi et al.2017 [61]	Case control	168	Italy	34.5±5.1	IADPSG	SAT: 107±4.8 mm VAT: 10.1±3.0 mm	24–28	• Age • BMI • Family history of diabetes	Significant	8
27	Han et al.2017 [62]	Cohort	17803	China	28.5±2.8	IADPSG	WC: 82.8±9.7 cm	4–12	• BP • BMI	Significant	7
28	He et al. 2017 [63]	Case control	255	China	29.1 ±3.7	ADA	NC: 35.20 ±2.56 cm WC: 103.16±8.00 cm	16	• Age • Gravidity • HbA1c • Lipid profile • BMI	Significant	7
29	Li et al.2017 [64]	cohort	371	china	31.0±3.0	IADPSG	NC: 34.3±1.5 cm	11–13	• Age • PPBMI • Lipid profile	Significant	7
30	Yang et al.2017 [65]	cohort	333	Korea	32±3.9	National Diabetes Data Group	SFT:2.7±0.6 cm	10–13	• Age • PPBMI • GWG	Significant	7
31	Alptekin et al.2016 [66]	Cohort	227	Turkey	28.8 ± 4.8	Carpenter and Coustan	WC: 89.7 ± 11.9 cm HC: 105.8 ± 14.2 cm WHR: 0.84 ± 0.04	7–12	• HOMA-IR • BMI WGDP	Significant	8
32	Basraon et al.2016 [67]	Cohort	2300	USA	23.3±4.9	Guidelines of each clinical center	WHR: 0.88 ± 0.07	9–16	• IR • BMI • Ethnicity	Significant	8
33	White et al.2016 [68]	Cohort	1303	UK	32.0 ±4.9	IADPSG	• NC: 37.4 ±2.5 cm • WC: 110 (103–116) cm • MUAC:37 (35–40) cm • HC: 123 (116–130) cm • WHR: 0.89 ±0.07	15–18	• Age • BP • Ethnicity • Parity • IR • Previous GDM • HgbA1C -Adiponectin • Sex hormone binding globulin • Triglycerides • PCOs • Smoking	Significant	8
34	De Souza et al.2015 [69]	Cohort	485	Canada	32.9 ±4.8	IADPSG	• SAT: 1.9± 0.80 cm • VAT: 4.1±1.7 cm • TAT: 5.9±2.1 cm	11–14	• AgeSi • BMI	Significant for TAT & VAT	
35	Kennedy et al.2015 [70]	Cohort	1350	Canada	29.3 ± 5.1	NR	• SAT1: 21.2 mm (6.9– • 73.9) SAT2: 20.3 mm (7.5–68.0)	11–14 (SAT1) 18–22 (SAT2)	• BMI	Significant	7
36	Sina et al.2015 [71]	Case control	131	Australia	23.7 ±5.5	ICD-9 and ICD -10	▪ WC:90.3 ±16.4 cm ▪ HC: 98.3 ±16.3 cm ▪ WHR: 0.92 ±0.05	-	• BMI	Significant for WC and HC	9
37	Balani et al.2014 [72]	Case control	302	UK	32.1±5.5	WHO	▪ WHR: 1.02±0.07 ▪ TPBF: 49.8±3.5 ▪ VAT: 199.2±40.5	14–17	• BMI	Significant for BMI, WHR, VFM	7
38	Bolognani et al.2014 [73]	Cross sectional	240	Brazil	17–40	WHO	WC: 93.548±8.873 cm	20–24	• PPBMI • BMI • GWG	Significant	8
39	Gur et al. 2014 [74]	Cohort	94	Turkey	43.4	WHO	WC:65.3 cm minimum subcutaneous fat (S_min_): 66.7 mm maximum pre-peritoneal visceral fat (V_max)_:67.2 mm	4–14	• BMI • FBG • Metabolic • syndrome • Lipid profile • BP • HOMA-IR Smoking	Significant	8
40	Mameghani et al.2013 [75]	Cohort	1140	Iran	17–40	WHO	WC: 81.84 ± 0.35 cm	<12	• BMI	Significant	8
41	Suresh et al.2012 [76]	Cohort	1200	Australia	17–45	The Royal Australian and New Zealand College of Obstetricians and Gynaecologists. C-Obs guideline	-SAT: 18.2 mm (range 6.3–50.9 mm)	18–22	• BMI	Significant	8

**ICD9**: International Classification of Diseases, 9th Revision-Clinical Modification, **H:** height**, WGDP:** weight gained during pregnancy**, HOMA-IR**: homeostasis model assessment insulin resistance**, WHR:** Waist/Hip Ratio**, QUICKI:** quantitative insulin sensitivity check index**, VAD:** Visceral Adipose Tissue Depth**, BMI**: Body Mass Index**, VFM:** visceral fat mass**, PBF:** percentage body fat**, IR**: insulin resistance, **WC:** waist circumference, **SAT:** subcutaneous tissues thickness, **TAT:** total adipose tissues thickness, **VAT:** visceral tissues thickness, **ASFT:** abdominal subcutaneous fat thickness, **FBG:** fasting blood glucose, **NC:** Neck circumference, **ISI:** insulin sensitivity index, **ASM:** appendicular skeletal muscle mass, **FM**: fat mass, **HbA1c:** glycosylated hemoglobin A1c,**SFT:** subcutaneous fat thickness, **IADPSG:** International Association of Diabetes and Pregnancy Study Groups, **FMP:** fat mass percentage, **SMMP:** skeletal muscle mass percentage, **FMI:** Fat mass index, **BFI:** Body Fat Index = **(pre-peritoneal fat x subcutaneous fat/height)**, **FFM:** fat free mass, **MM:** muscular mass, **PP**: Pre pregnancy, **PPBMI:** Pre pregnancy BMI, **ADA**: American Diabetes Association, **WHO**: World health Organization, **ACOG**: American College of Obstetricians and Gynecologists, **AC**: arm circumference, **NS**: Not Significant

*: OR

**: median (IQR)

***: AUC (CI)

****: median (max-min)

## Discussion

The current study set to investigate the relationship between body composition and GDM as a systematic review and meta-analysis. The results indicate that anthropometric indices such as WC, NC, HC, WHR, VAT, SAT, Height, and MUAC are associated with GDM; an increase in the indices of WC, NC, HC, WHR, VAT, SAT, and MUAC increase developing GDM, also short stature increases the susceptibility to GDM.

We investigated that VAT and SAT are associated with GDM. Alwash et al.(2021) found that all three obesity phenotypes were significantly associated with the risk of developing GDM. In addition, visceral obesity was a stronger risk factor for GDM than other obesity phenotypes [32]. Yao et al.(2020) also stated that the risk of GDM is associated with maternal central obesity in early pregnancy [77]. In the case of central and visceral body fats, Benevides et al.(2020) reported that the cut-off point for subcutaneous, visceral, and total abdominal fat to predict GDM varied between studies in the first and second trimesters of pregnancy. No study confirmed a model for predicting GDM using subcutaneous and visceral fat measurements [78].

De Souza et al.(2015) determined the relationship between SAT depth, TAT depth, and VAT depth in the first trimester of pregnancy and the occurrence of GDM in mid-pregnancy. It was observed that increasing the depth of VAT and TAT independently of BMI could predict the risk of dysglycemia in later stages of pregnancy [69]. Similarly, Balani et al. (2018) showed that visceral adipose mass in obese women can be a predictor of GDM [57]. Increased VAT depth, but not SAT depth, was associated with an increased risk of GDM after adjusting for confounding factors. VAT depth ≥ 4.27 cm is more sensitive compared to the National Institute of Health and Care Excellence criteria and similar feature for the diagnosis of GDM [79]. In addition, Alves et al.(2020) observed an increase in VAT depth in sonographic measurements in early pregnancy; GDM was associated with a higher risk [28]. One of the strengths of the present study is the assessment of most indices of body composition and their relationship with GDM and the large number of up-to-date studies that lead to the investigation of more samples.

The results of the present study also showed WC, HC and WHR are associated with GDM. Various studies have shown an association between WC and WHR-based central obesity around the hip with the occurrence of GDM [31]. However, the data are also contradictory; for example, Basraon et al.(2016) showed that WHR could not replace BMI as a risk factor in pregnancy for GDM [67]. But, Yao et al.(2020) in his subgroup analysis showed that higher levels of central maternal obesity in the first stage have a similar risk of GDM in the first and second trimesters of pregnancy [77]. However, Tornaghi et al.(1994) provided evidence of the superiority of maternal central obesity regarding mid-pregnancy (18–22 weeks) in identifying obesity-related complications in pregnancy. In other words, the factors expressing central obesity in the mother’s body can better predict the risk of GDM than BMI [80]. Central obesity is expressed as a risk factor for insulin resistance associated with deposition and abnormal fat function. WC as one of the indices of central obesity leads to an increased risk of GDM. Multivariate regression analysis with consideration of other risk factors showed that WC ≥ 80 cm could not predict the risk of GDM. However, Ebrahimi-Mameghani et al.(2013) concluded that WC≥88 cm is a significant predictor of GDM (OR: 3.77) [41, 75]. Han et al. (2018) also observed that the risk of GDM increases with WC≥78.5 cm increase [75]. WC at gestation weeks 20–24, pre-pregnancy BMI, and gestational BMI can predict the occurrence of GDM. WC 100 cm with 84% sensitivity and 70% specificity predicts GDM risk [50]. Although other studies have shown that at gestation weeks 20–24, WC: 85.5–88.5 cm was the optimal cut-off point for GDM prediction (Sens/Spec balance between 87.1/41.1% and 77.4/56.9%) [73].

Kansu-Celik et al.(2018) observed a significant relationship between 50g GCT and WC, and SAT thickness. He showed that SAT predicts thickness greater than 16.75 mm GDM with a sensitivity of 71.7% and a specificity of 87.6% [40]. In adults, WHR is independently associated with complications after relative weight adjustment, i.e. the use of relative weight and body shape at the same time provides a better estimate of the risk of disease than either alone [81]. In women with WHR<0.85, one or more risk factors increased the risk of GDM by 1.99 times, and in women with WHR≥0.85 but without fixed risk factors, the risk of GDM increased by 2.41 times, and in women with fixed risk factors, it increased by 6.22 times. Similar but weak results were observed for WC≥88 cm [31].

We have shown that increased NC also leads to GDM. Hancerliogullari et al.(2020) also stated that NC in women with GDM are significantly higher [29] and NC is assumed to be a better marker than WC for determining metabolic syndrome and its key features. It is also easy to measure and it is replicable [82, 83]. Barforoush et al.(2021) also stated that NC more than 34.3 cm in Iranian women could predict GDM [46].

In this study we reported that short stature increases the susceptibility to GDM. Height in adulthood is an indices of genetic, early and childhood factors and their interactions. Although the biological mechanism associated with adult height and GDM is unknown, several pathways have been suggested. For example, malnutrition of the fetus may lead to low birth weight, which is associated with shorter height in adulthood, and may also be associated with metabolic disorders in adulthood. Height has different variations in different populations [84, 85]. In an analysis of 135861 pregnant women, height was found to be inversely related to the occurrence of GDM. Of course, this relationship can also vary between different races [86].

Body composition in pregnancy has a dynamic process; for example, changes in weight gain and free body adipose mass during pregnancy are clearly observed [87].

Measuring maternal body composition during pregnancy is challenged by existing in-vivo measurement methods that cannot distinguish between maternal and fetal reserves [88] and look at the mother and fetus as a whole. In addition, some pregnancy-induced changes in body composition violate the assumptions that underlie many commonly available measurement methods and require special pregnancy modifications (which often vary at different gestational ages) [89].

The composition of the mother’s body changes during pregnancy to support optimal fetal growth. In the first few months of pregnancy, changes in the composition of the mother’s body indicate the readiness of the female body for fetal growth. Especially, the uterine and breast tissue that makes up the mother unit grows and the blood volume increases. In late pregnancy, more pronounced growth of the embryonic unit (including the fetus, amniotic fluid, and placenta) occurs along with the continued growth of maternal tissue and further increase in blood volume. At the time of delivery, the fetal unit accounts for approximately one-third of the total GWG [90].

Accordingly, central obesity is associated with more obesity-related complications [91]. In contrast, peripheral obesity has been suggested to eliminate or even protect against some of the risks associated with obesity [92]. CT, MRI, body densitometry, or WHR are better indices of central obesity than BMI but are impractical as screening tools in pregnancy. SAT measurement can be used as an alternative measure of central obesity [93] as it is associated with a wide range of cardiovascular and metabolic risk factors. SAT can be easily and accurately measured by ultrasound [94]. BMI can also be potentially useful as a direct and inexpensive method for assessing central fat distribution [95]. In adults, BMI can predict outcomes such as type-2 diabetes and hypertension[81]. Although a sufficient number of studies examining the relationship between BMI and GDM have been performed in the past [96, 97].

## Conclusion

Body composition indices such as WC, HC, WHR, AC, VAT, SAT, and height can relate more effectively and accurately to GDM. These available anthropometric indices can be used as a tool to assess the occurrence of GDM in an accessible, cost-effective, and high-precision manner.

### Limitation

One of the limitations of the study is the difference in the critical values of the criteria used to diagnose GDM, which may affect the decision on the absence or occurrence of GDM based on different indices. In addition, studies conducted in different populations and races, which is a determining factor in body composition and can affect both body composition and the occurrence of GDM, have not been considered in the present study. Also, the small number of studies performed on some anthropometric indices is another limitation of the study, which makes it difficult to draw conclusions about such indices.

## Supporting information

S1 File Forest plot of different anthropometric indices between GMD and non-GDM group. (DOC)

S1 Checklist PRISMA-2009-checklist. (DOC)

## Abbreviations

GDM: gestational diabetes mellitus
GWG: gestational weight gain
ICD9: International Classification of Diseases, 9th Revision-Clinical Modification
H: height
WGDP: weight gained during pregnancy
HOMA-IR: homeostasis model assessment insulin resistance
WHR: Waist/Hip Ratio
QUICKI: quantitative insulin sensitivity check index
VAD: Visceral Adipose Tissue Depth
BMI: Body Mass Index
VFM: visceral fat mass
PBF: percentage body fat
IR: insulin resistance
WC: waist circumference
SAT: subcutaneous tissues thickness
TAT: total adipose tissues thickness
VAT: visceral tissues thickness
ASFT: abdominal subcutaneous fat thickness
FBG: fasting blood glucose
NC: Neck circumference
ISI: insulin sensitivity index
ASM: appendicular skeletal muscle mass
FM: fat mass
HbA1c: glycosylated hemoglobin A1c
SFT: subcutaneous fat thickness
IADPSG: International Association of Diabetes and Pregnancy Study Groups
FMP: fat mass percentage
SMMP: skeletal muscle mass percentage
FMI: Fat mass index
BFI: Body Fat Index = (pre-peritoneal fat x subcutaneous fat/height)
FFM: fat free mass
MM: muscular mass
PP: Pre pregnancy
PPBMI: Pre pregnancy BMI
ADA: American Diabetes Association
WHO: World health Organization
ACOG: American College of Obstetricians and Gynecologists
AC: arm circumference
MUAC: mid upper arm circumference
NS: Not Significant
