# Supplementary material for: Association of body composition in early pregnancy with gestational diabetes mellitus: A meta-analysis
Source: PLoS One. 2022 Aug 15;17(8):e0271068. doi: 10.1371/journal.pone.0271068 (PMC9377632; doi:10.1371/journal.pone.0271068)
Supplement: S1 File — (DOC) [file pone.0271068.s001.doc]

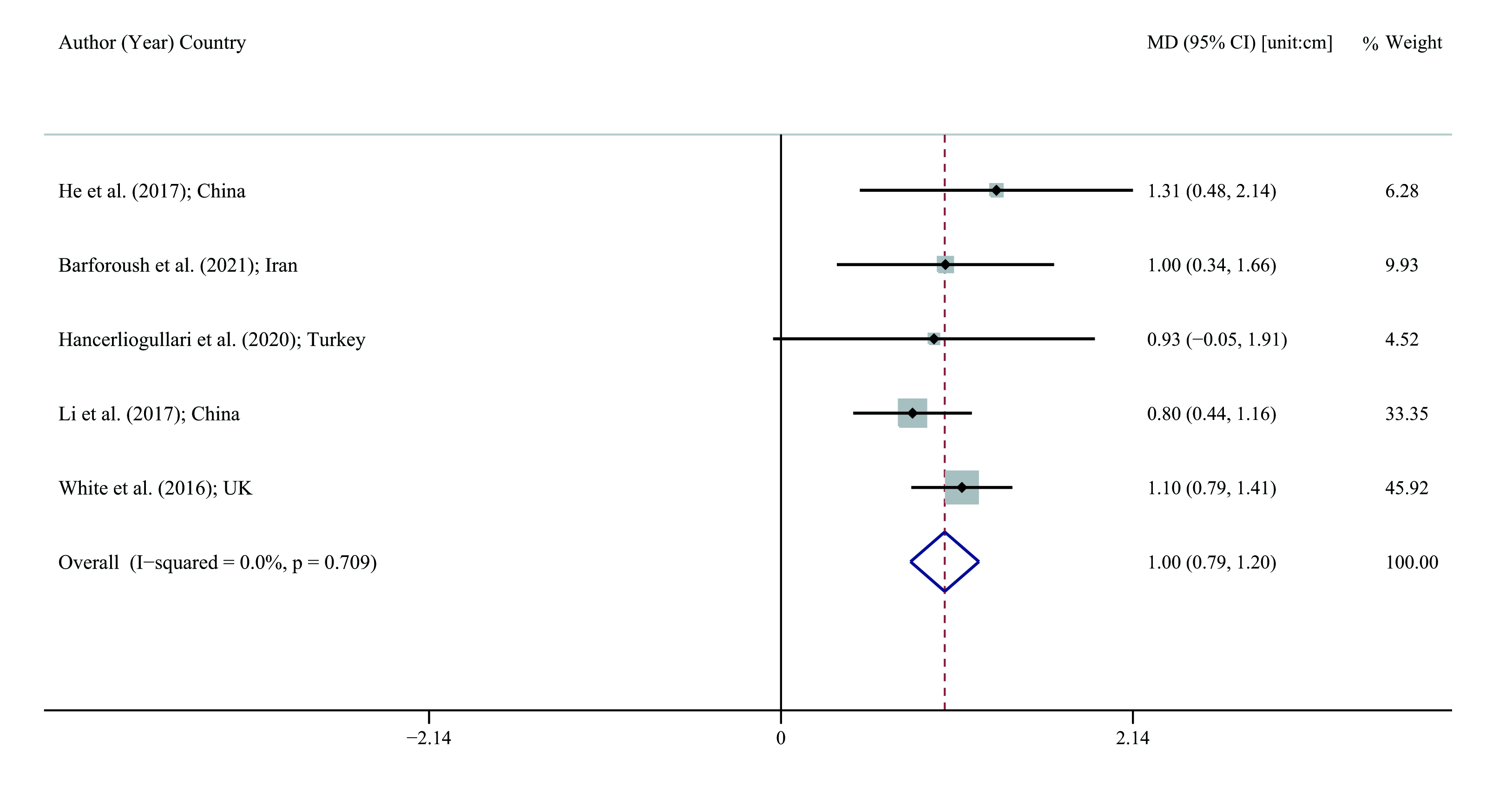


Supplement figure 1: forest plot for mean difference of neck circumference between GMD and non-GDM group. Each line segment's midpoint shows the mean difference estimate, length of line segment indicates 95% confidence interval (CI) in each study, and diamond mark illustrates the pooled estimate of mean difference.


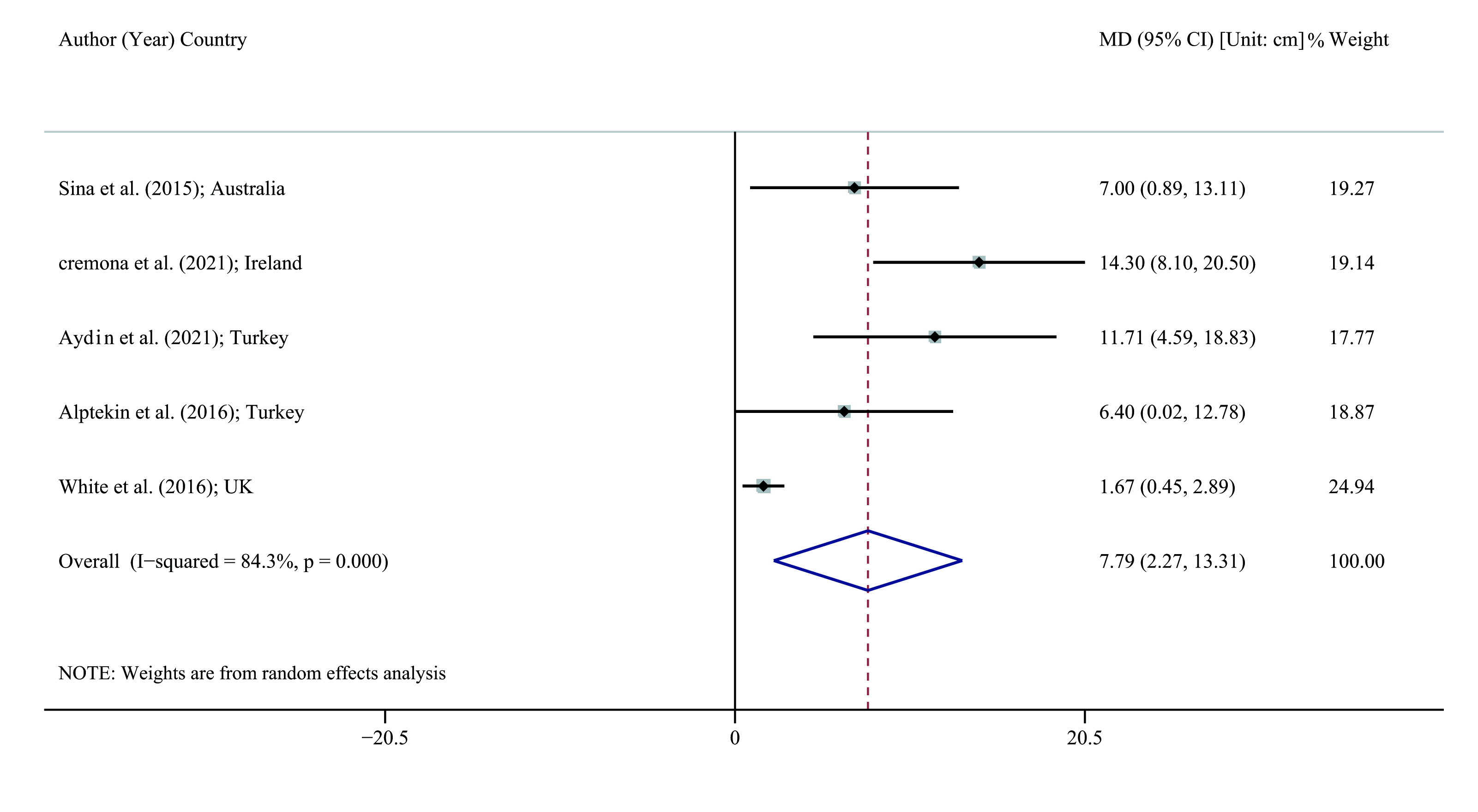


Supplement figure 2: forest plot for mean difference of hip circumference between GMD and non-GDM group. Each line segment's midpoint shows the mean difference estimate, length of line segment indicates 95% confidence interval (CI) in each study, and diamond mark illustrates the pooled estimate of mean difference.


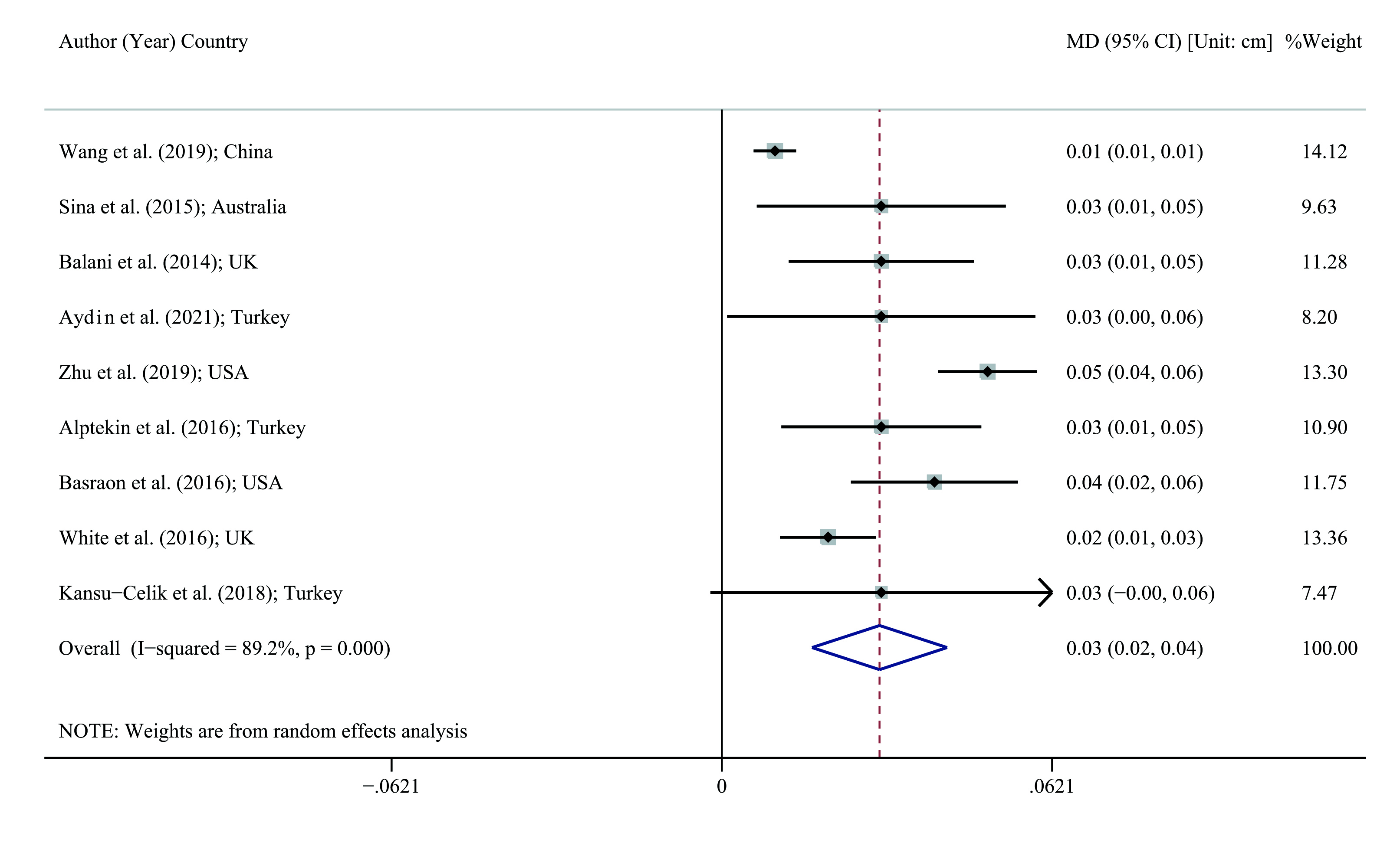


Supplement figure 3: forest plot for mean difference of Waist Hip Ratio between GMD and non-GDM group. Each line segment's midpoint shows the mean difference estimate, length of line segment indicates 95% confidence interval (CI) in each study, and diamond mark illustrates the pooled estimate of mean difference.


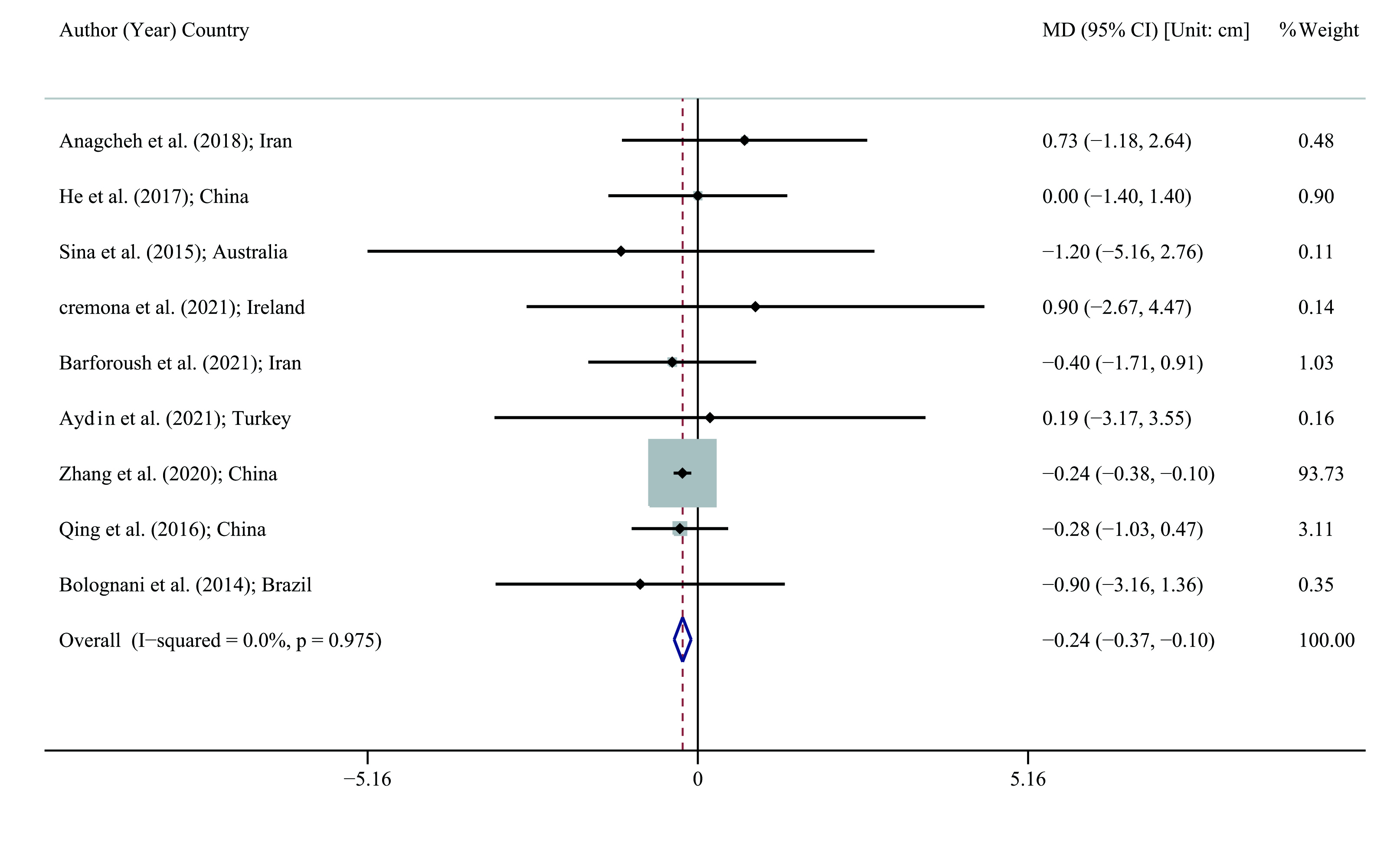


Supplement figure 4: forest plot for mean difference of Height between GMD and non-GDM group. Each line segment's midpoint shows the mean difference estimate, length of line segment indicates 95% confidence interval (CI) in each study, and diamond mark illustrates the pooled estimate of mean difference.


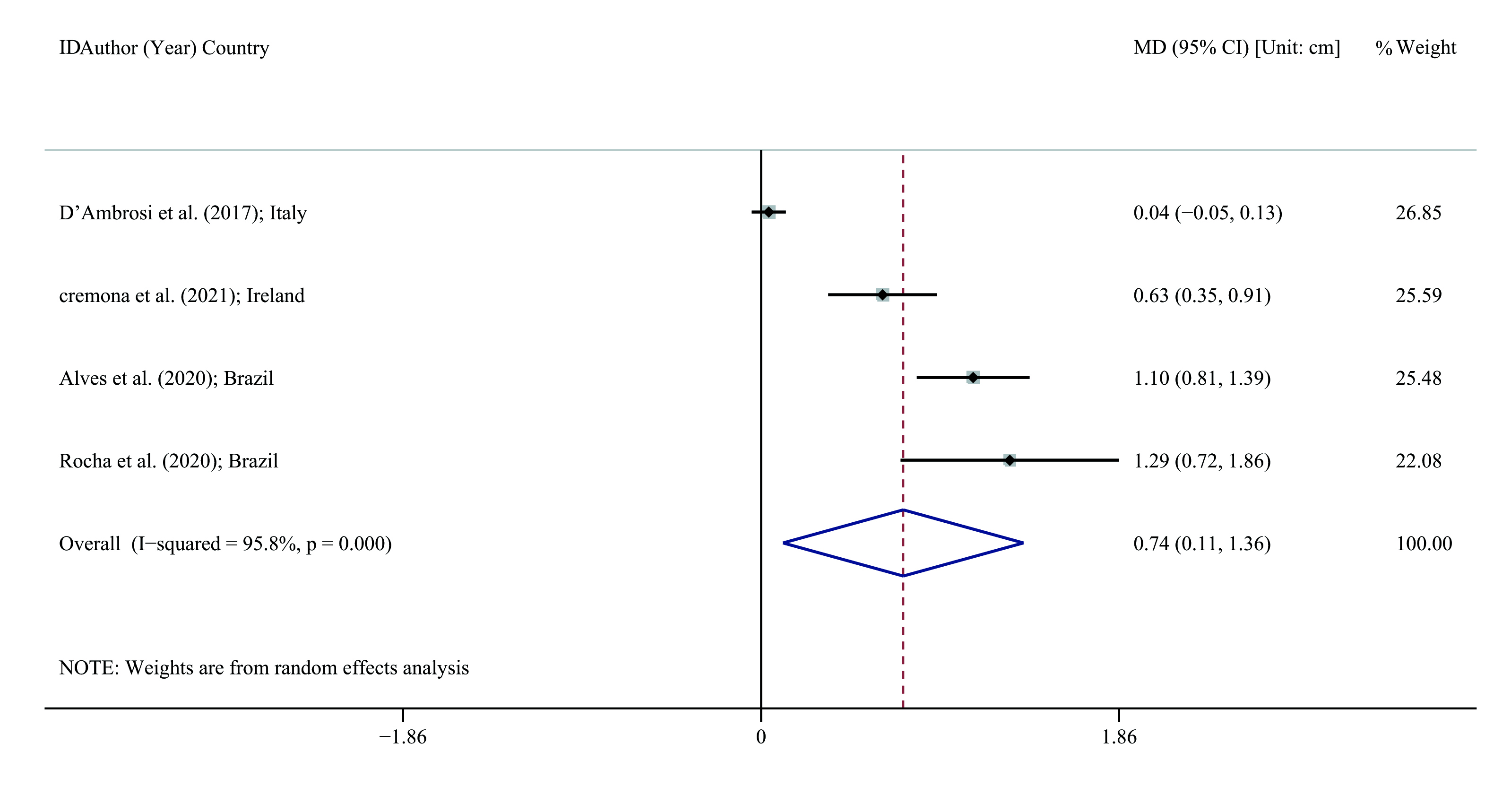


Supplement figure 5: forest plot for mean difference of Visceral Adipose Tissue Depth between GMD and non-GDM group. Each line segment's midpoint shows the mean difference estimate, length of line segment indicates 95% confidence interval (CI) in each study, and diamond mark illustrates the pooled estimate of mean difference.


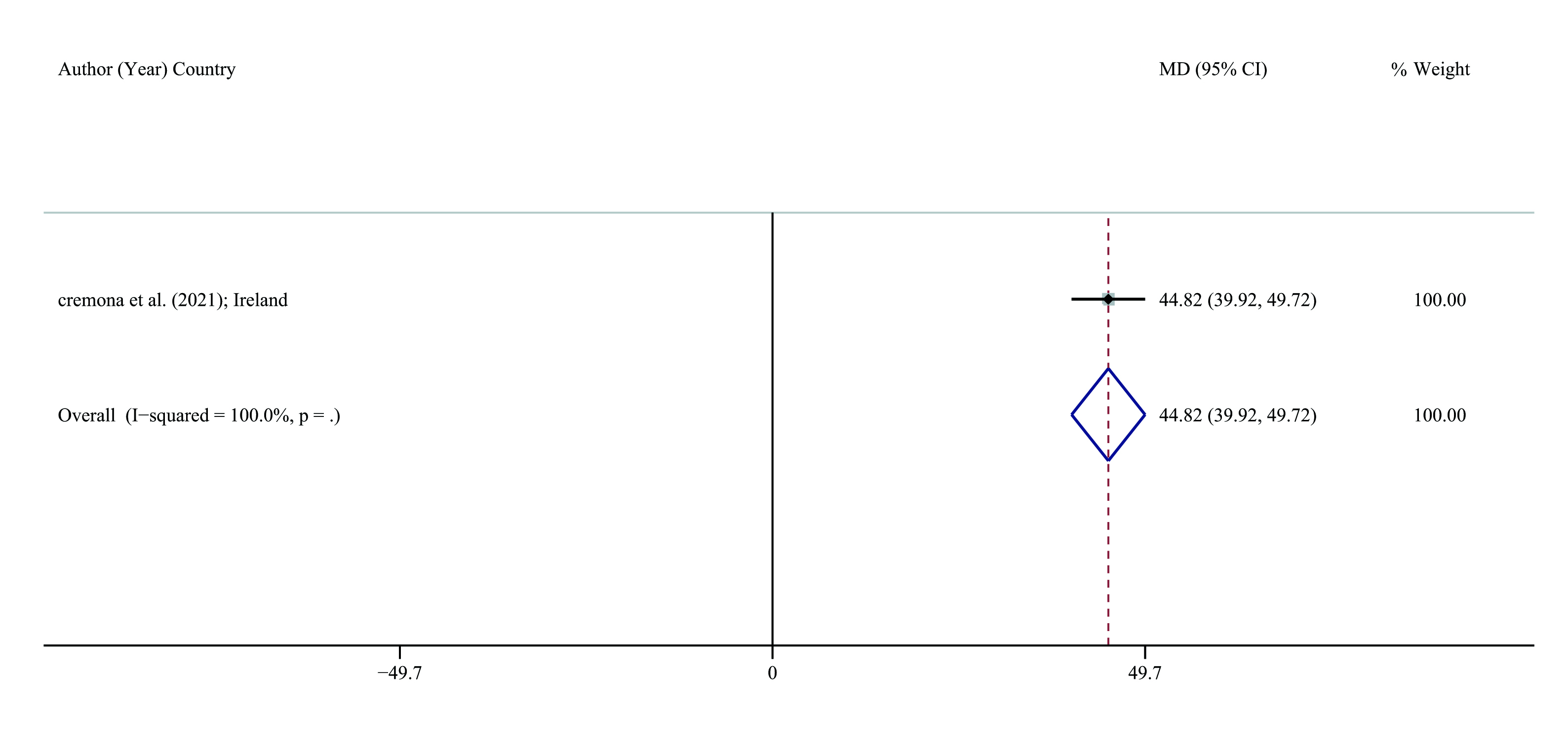


Supplement figure 6: forest plot for mean difference of Fat mass percentage between GMD and non-GDM group. Each line segment's midpoint shows the mean difference estimate, length of line segment indicates 95% confidence interval (CI) in each study, and diamond mark illustrates the pooled estimate of mean difference.


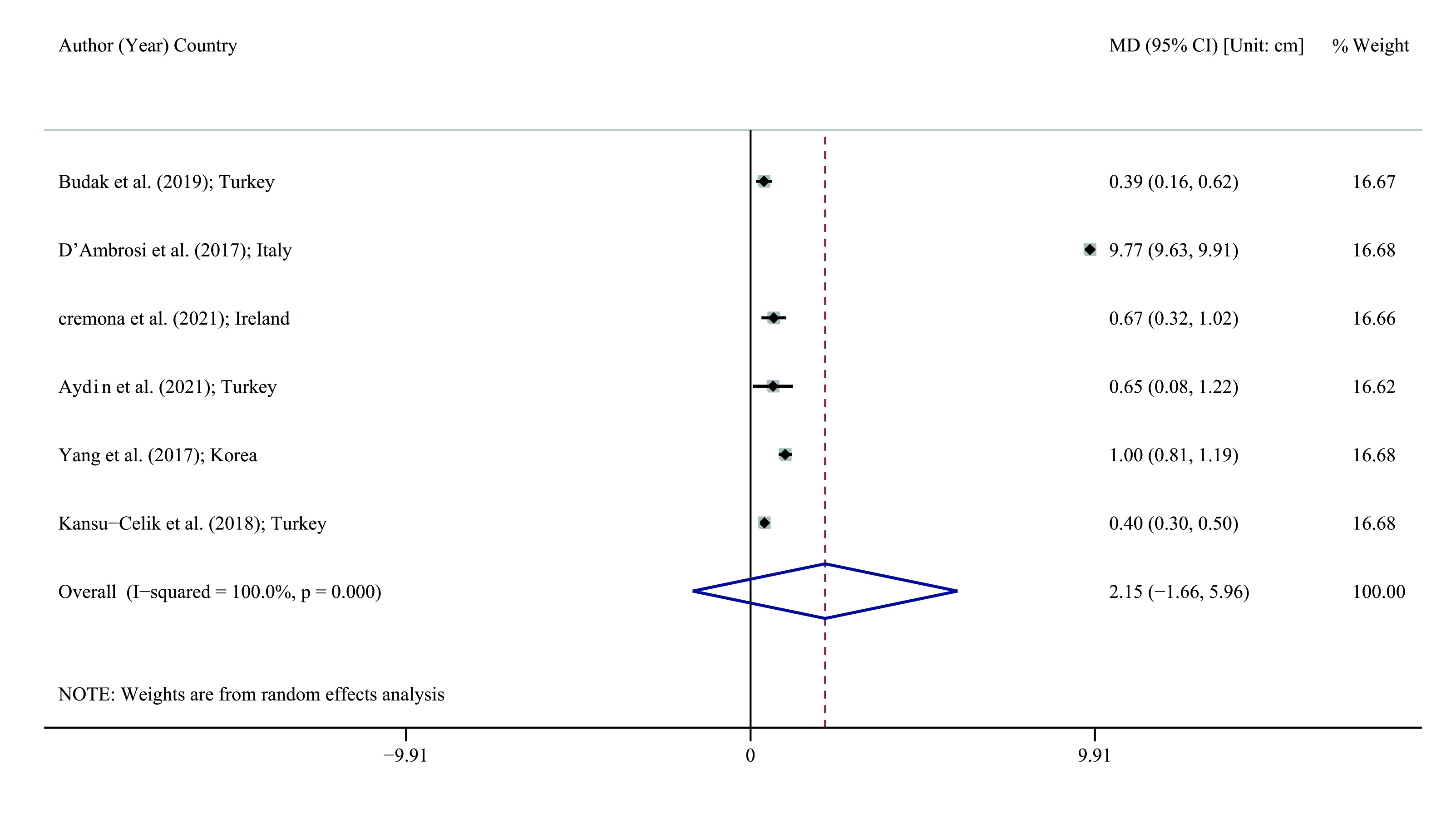


Supplement figure 7: forest plot for mean difference of Subcutaneous adipose tissues between GMD and non-GDM group. Each line segment's midpoint shows the mean difference estimate, length of line segment indicates 95% confidence interval (CI) in each study, and diamond mark illustrates the pooled estimate of mean difference.


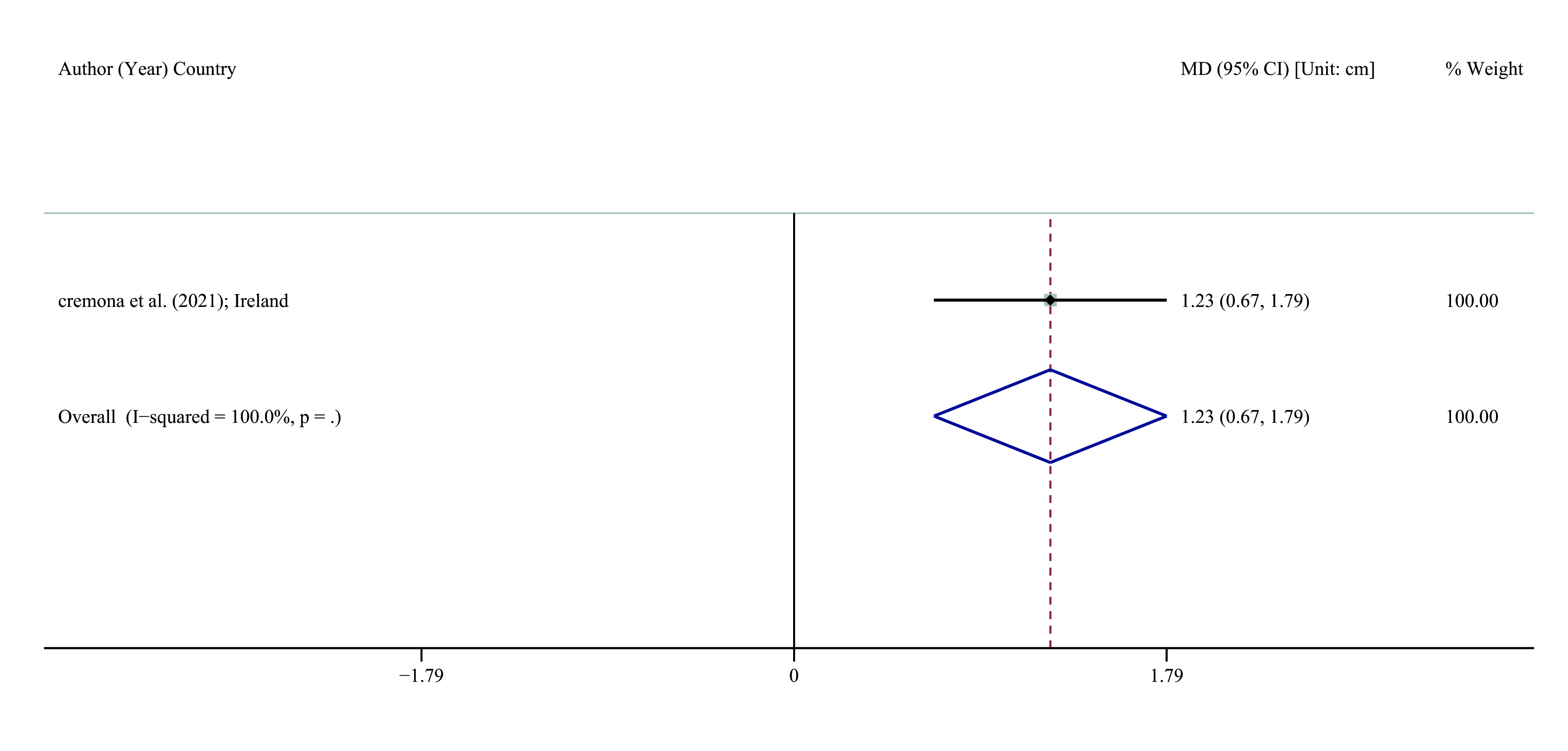


Supplement figure 8: forest plot for mean difference of Total adipose tissues thickness between GMD and non-GDM group. Each line segment's midpoint shows the mean difference estimate, length of line segment indicates 95% confidence interval (CI) in each study, and diamond mark illustrates the pooled estimate of mean difference.


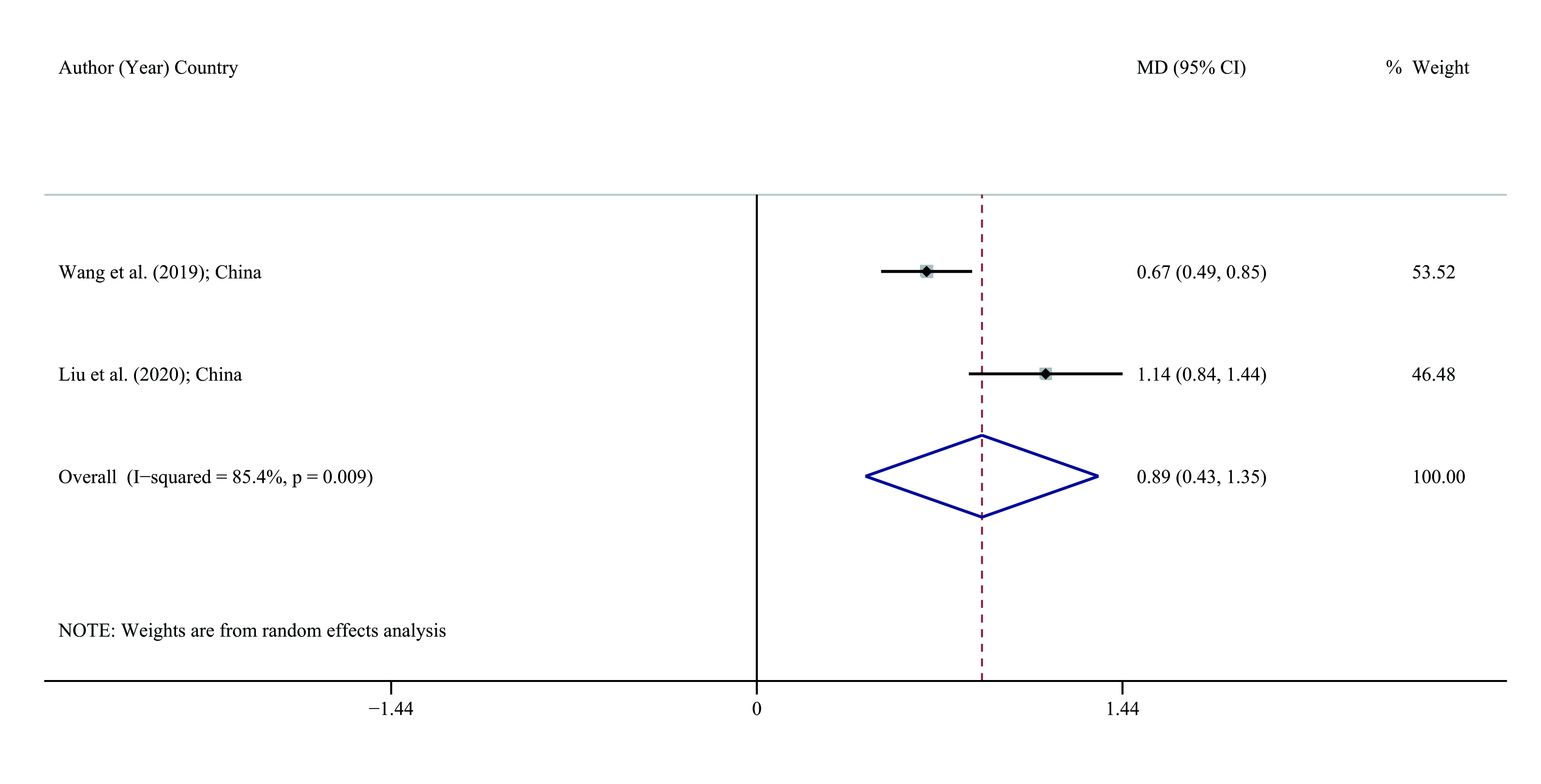


Supplement figure 9: forest plot for mean difference of Fat mass Index between GMD and non-GDM group. Each line segment's midpoint shows the mean difference estimate, length of line segment indicates 95% confidence interval (CI) in each study, and diamond mark illustrates the pooled estimate of mean difference.


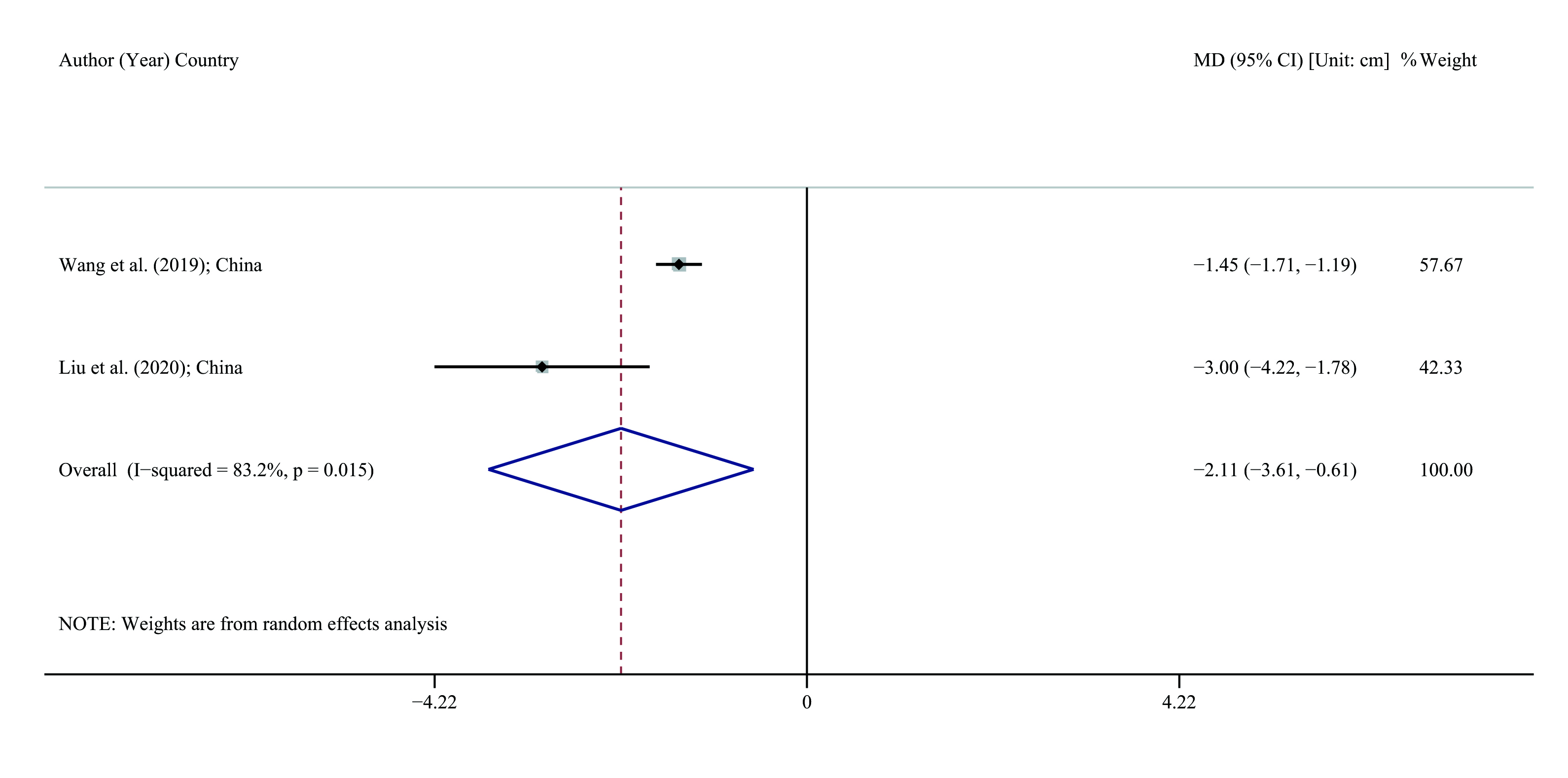


Supplement figure 10: forest plot for mean difference of Skeletal muscle mass percentage between GMD and non-GDM group. Each line segment's midpoint shows the mean difference estimate, length of line segment indicates 95% confidence interval (CI) in each study, and diamond mark illustrates the pooled estimate of mean difference.


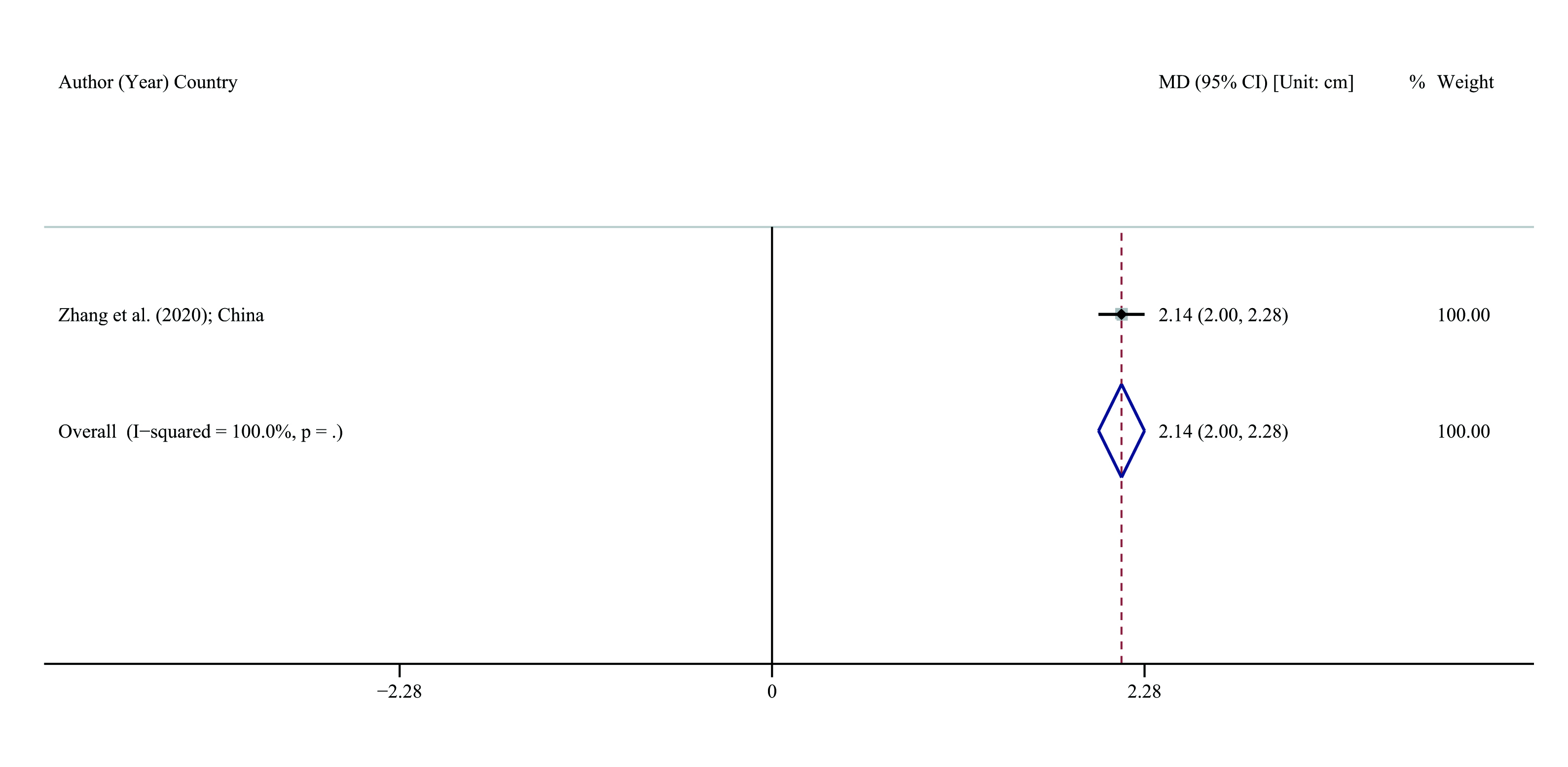


Supplement figure 11: forest plot for mean difference of Fat free mass between GMD and non-GDM group. Each line segment's midpoint shows the mean difference estimate, length of line segment indicates 95% confidence interval (CI) in each study, and diamond mark illustrates the pooled estimate of mean difference.


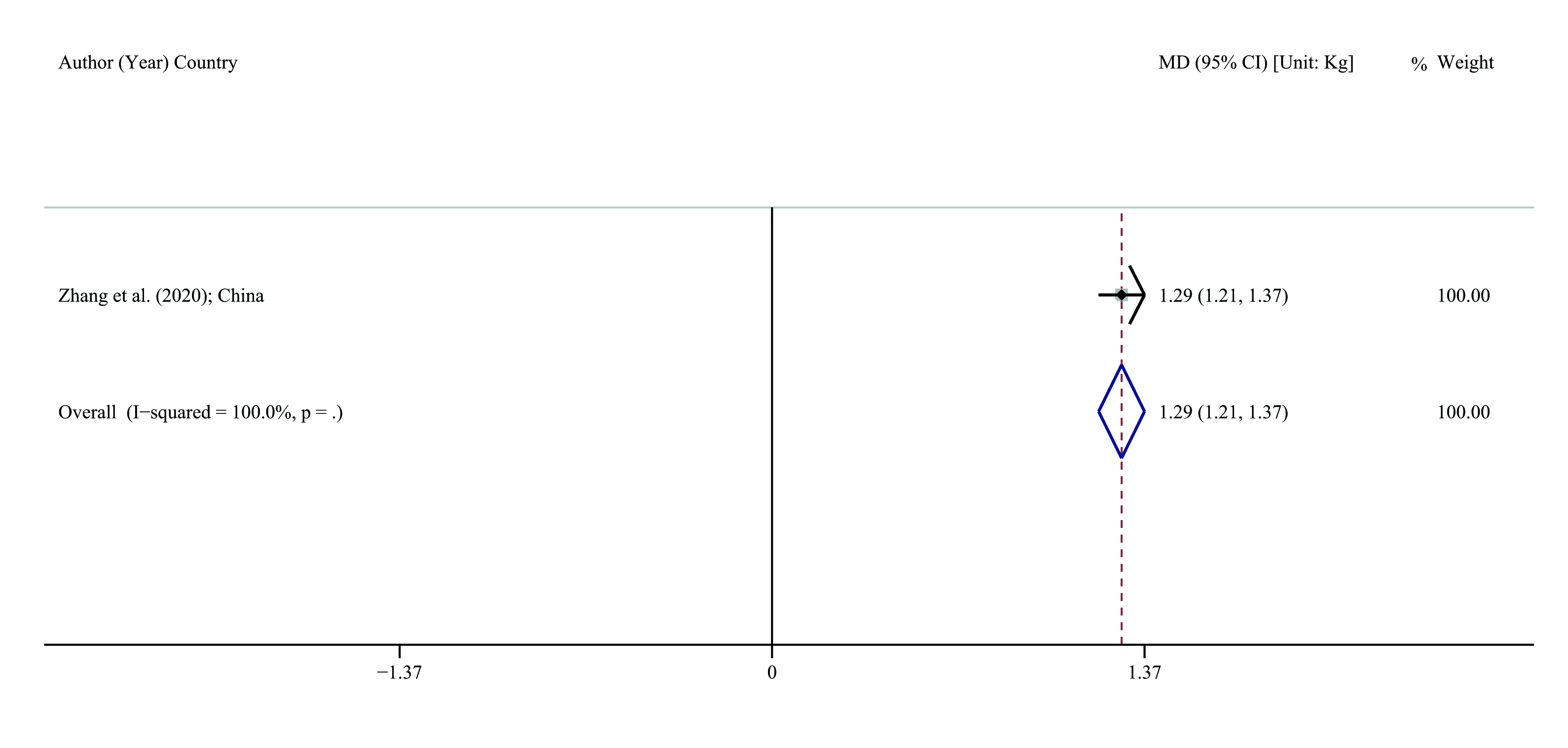


Supplement figure 12: forest plot for mean difference of Muscular mass between GMD and non-GDM group. Each line segment's midpoint shows the mean difference estimate, length of line segment indicates 95% confidence interval (CI) in each study, and diamond mark illustrates the pooled estimate of mean difference.


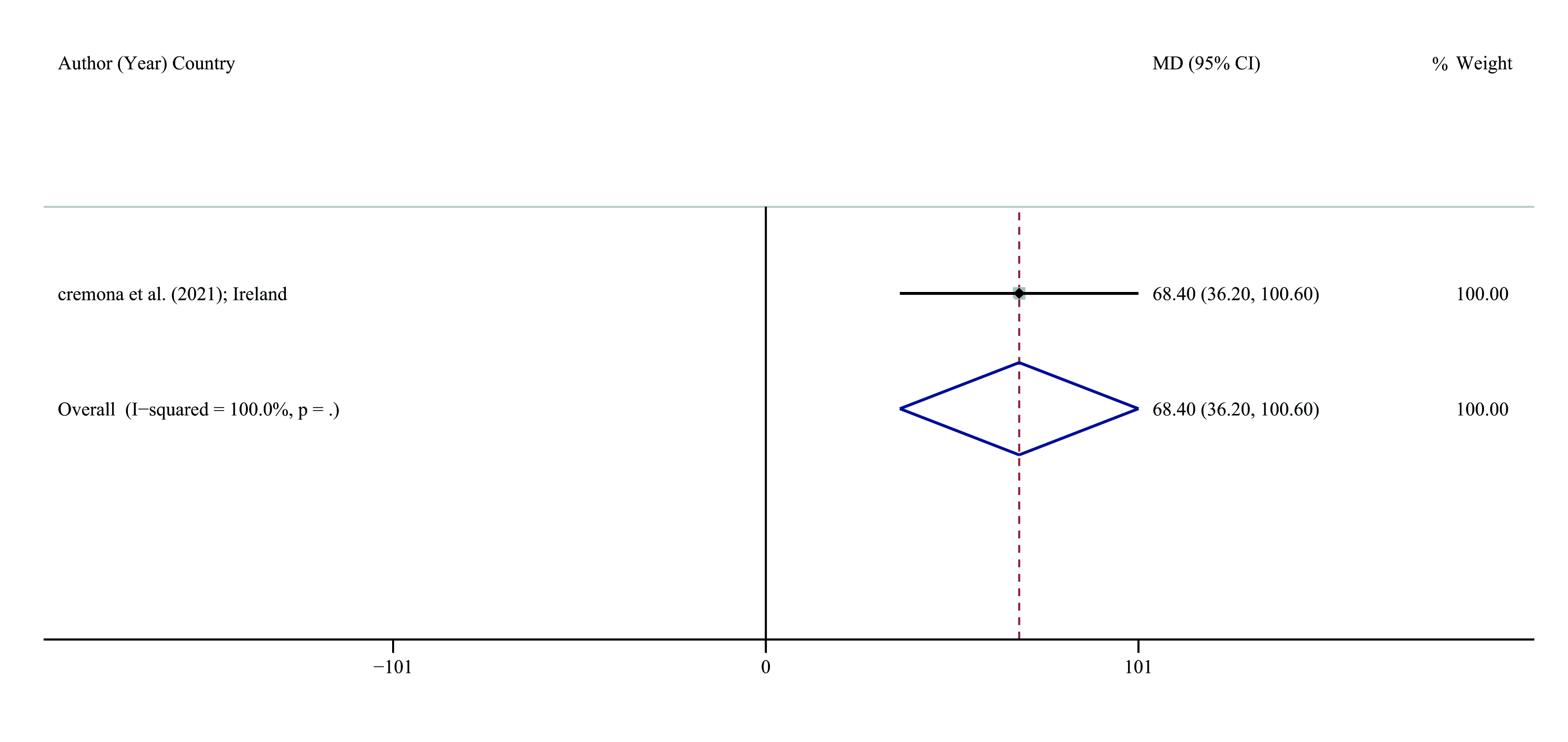


Supplement figure 13: forest plot for mean difference of Skin fold fat thickness between GMD and non-GDM group. Each line segment's midpoint shows the mean difference estimate, length of line segment indicates 95% confidence interval (CI) in each study, and diamond mark illustrates the pooled estimate of mean difference.


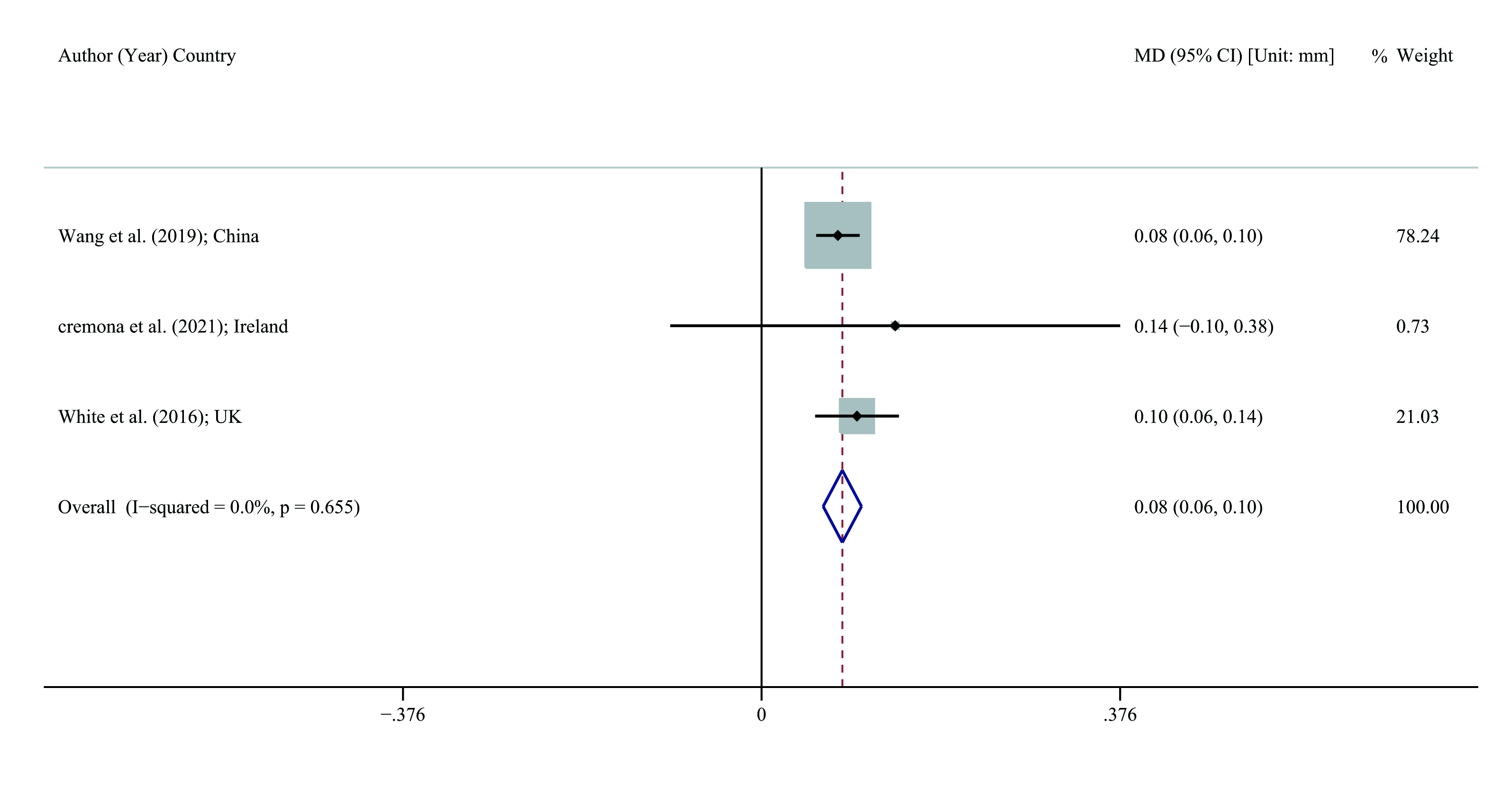


Supplement figure 14: forest plot for mean difference of Mid upper arm circumference between GMD and non-GDM group. Each line segment's midpoint shows the mean difference estimate, length of line segment indicates 95% confidence interval (CI) in each study, and diamond mark illustrates the pooled estimate of mean difference.


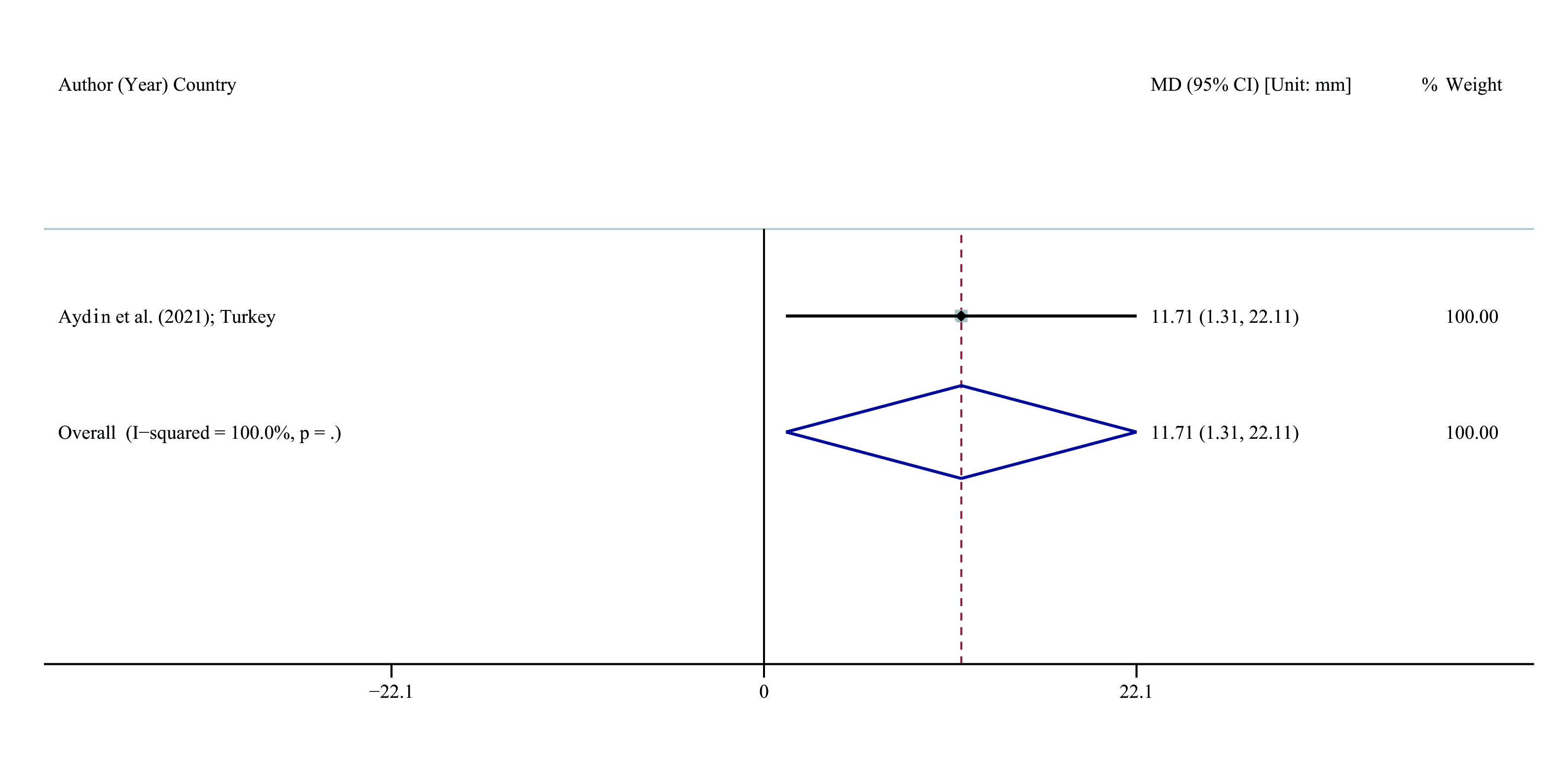


Supplement figure 15: forest plot for mean difference of Intra peritoneal fat thickness between GMD and non-GDM group. Each line segment's midpoint shows the mean difference estimate, length of line segment indicates 95% confidence interval (CI) in each study, and diamond mark illustrates the pooled estimate of mean difference.


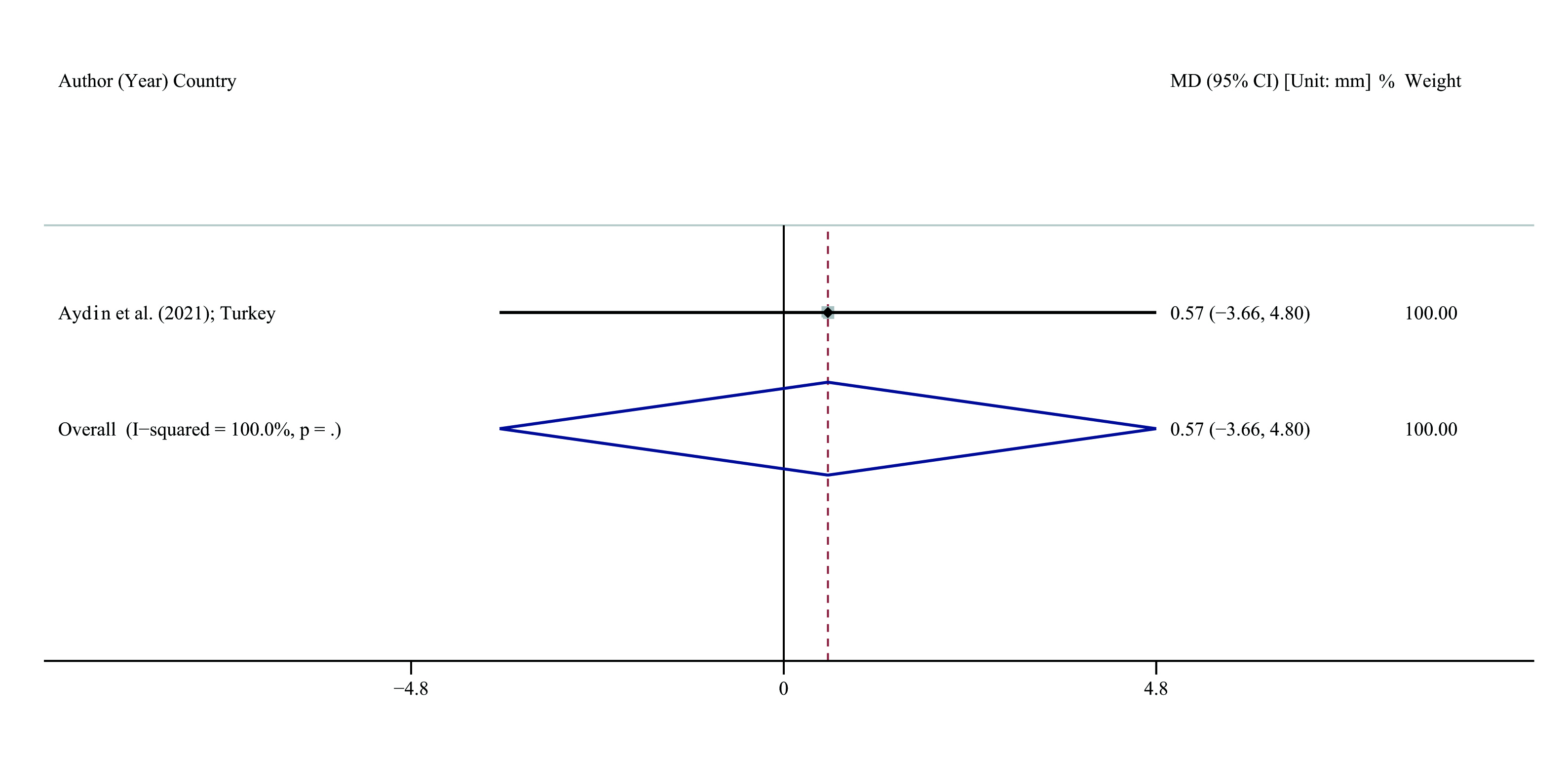


Supplement figure 16: forest plot for mean difference of Perirenal fat thickness between GMD and non-GDM group. Each line segment's midpoint shows the mean difference estimate, length of line segment indicates 95% confidence interval (CI) in each study, and diamond mark illustrates the pooled estimate of mean difference.


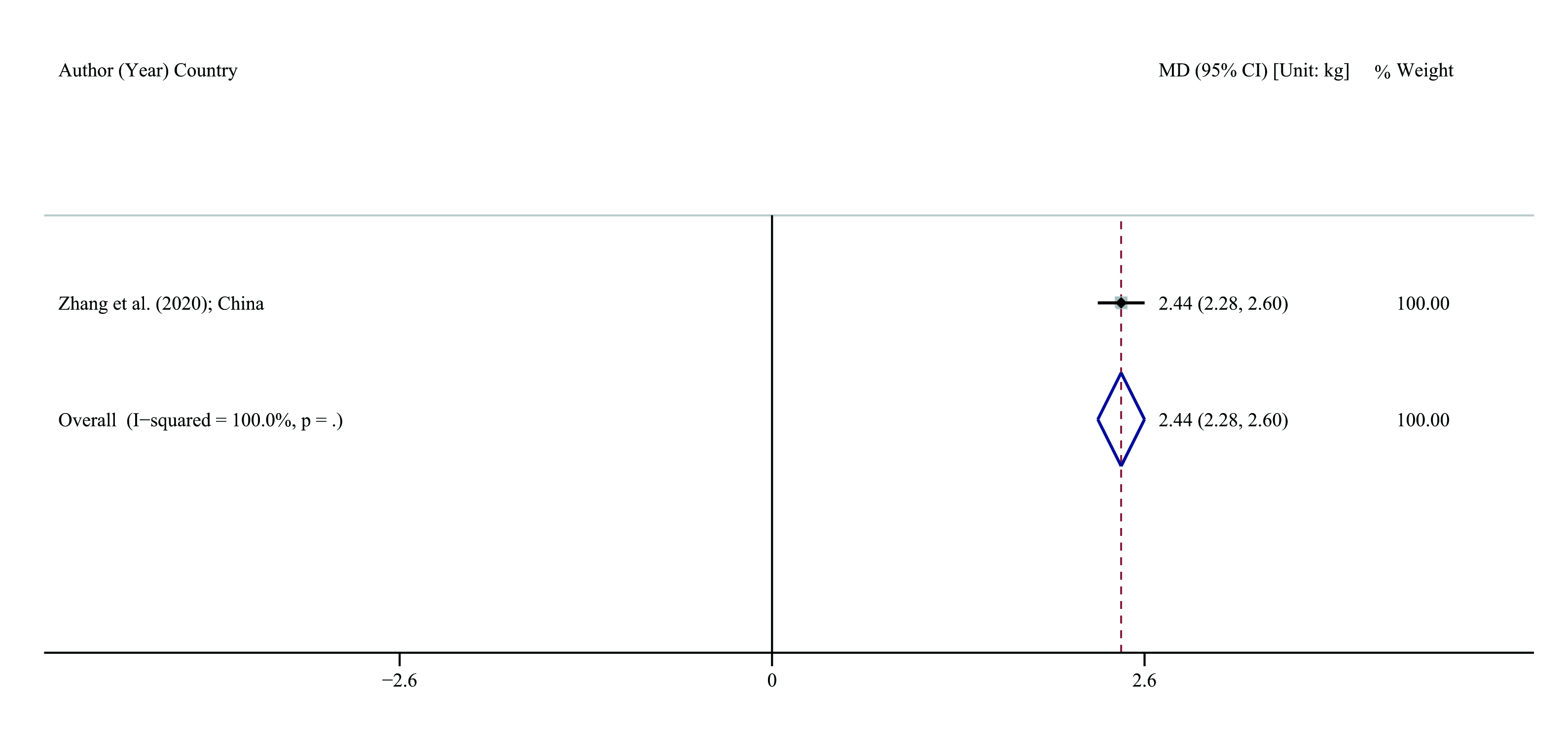


Supplement figure 17: forest plot for mean difference of Fat mass between GMD and non-GDM group. Each line segment's midpoint shows the mean difference estimate, length of line segment indicates 95% confidence interval (CI) in each study, and diamond mark illustrates the pooled estimate of mean difference.


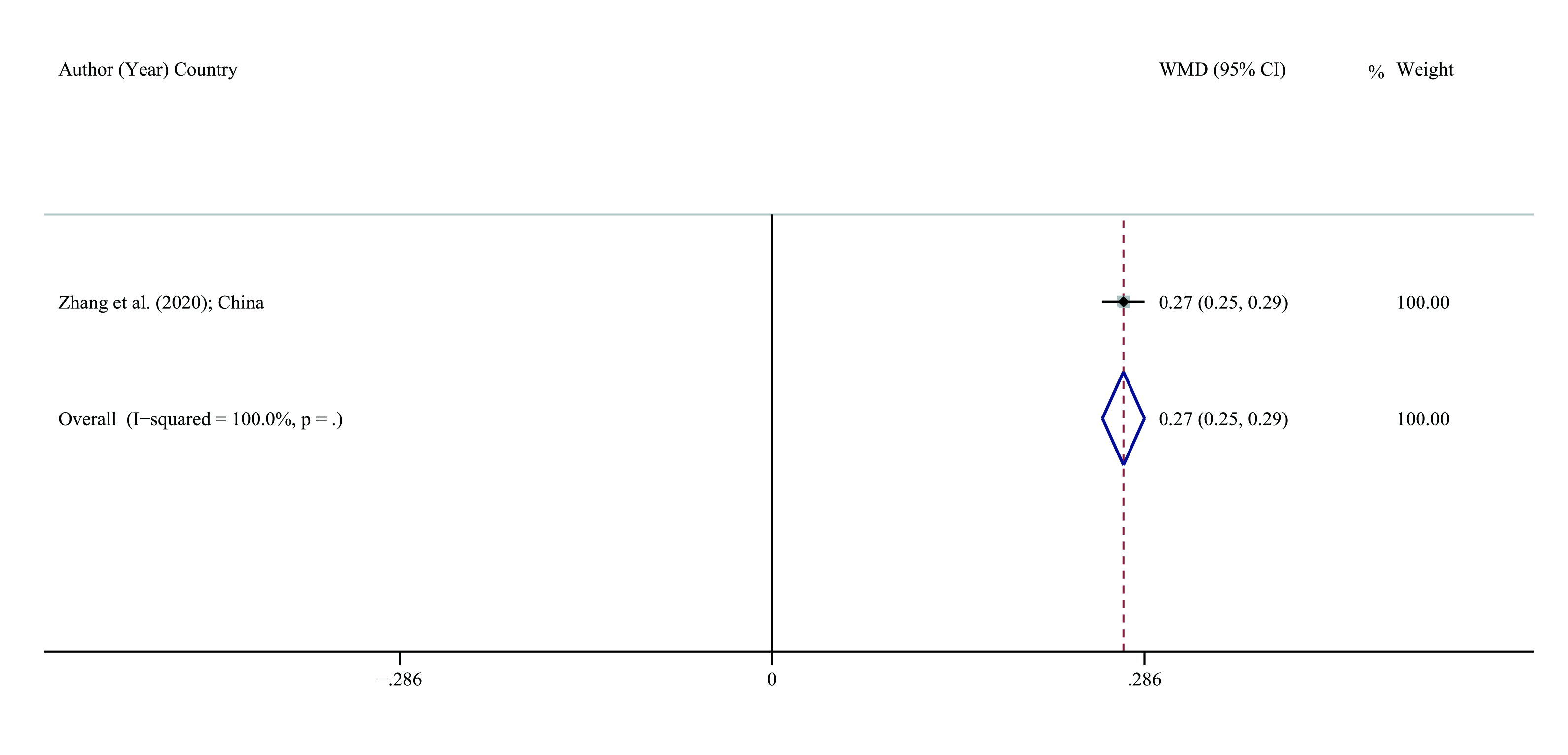


Supplement figure 18: forest plot for mean difference of Visceral fat level between GMD and non-GDM group. Each line segment's midpoint shows the mean difference estimate, length of line segment indicates 95% confidence interval (CI) in each study, and diamond mark illustrates the pooled estimate of mean difference.


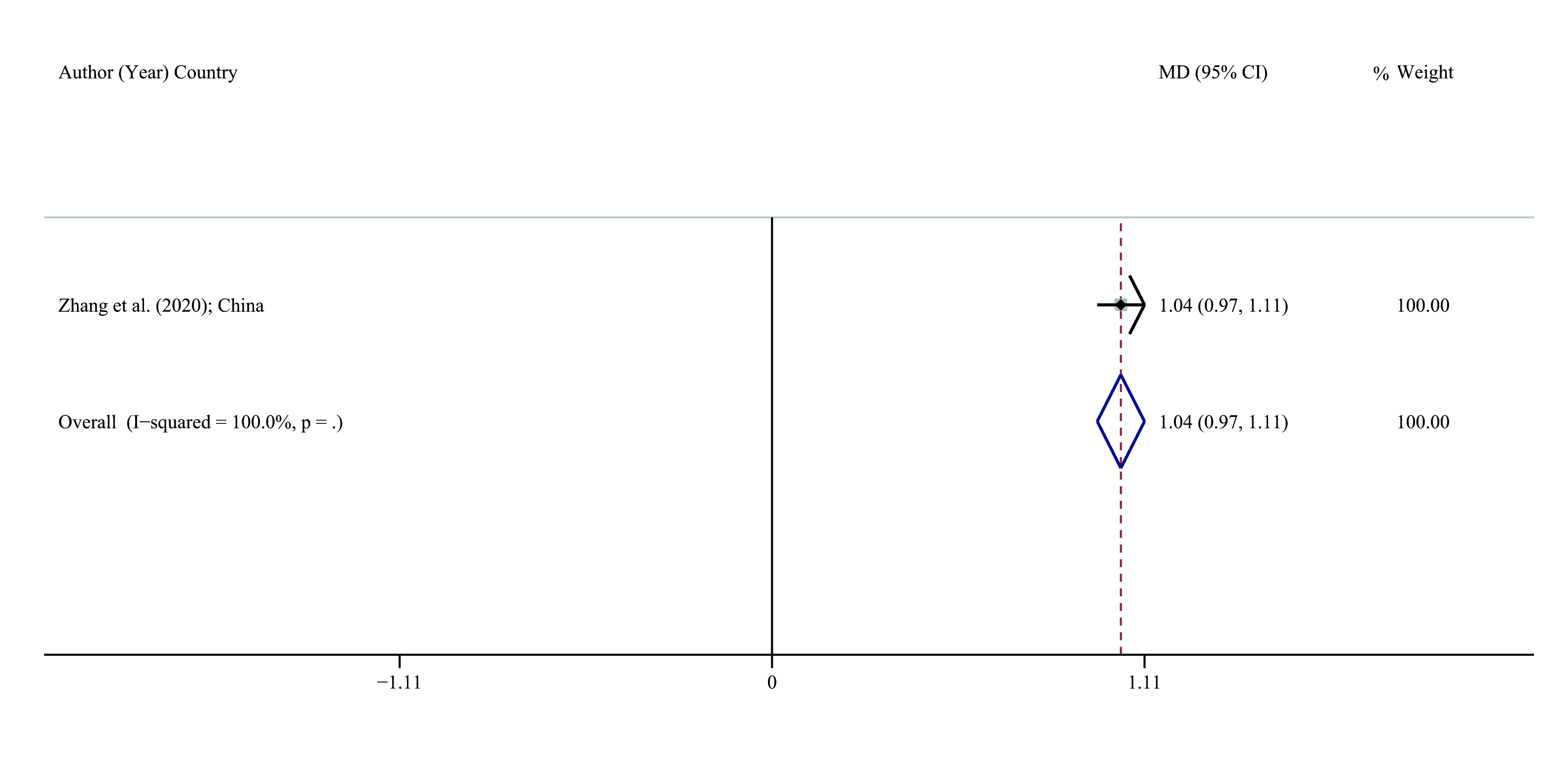


Supplement figure 19: forest plot for mean difference of Lean trunk mass GMD and non-GDM group. Each line segment's midpoint shows the mean difference estimate, length of line segment indicates 95% confidence interval (CI) in each study, and diamond mark illustrates the pooled estimate of mean difference.


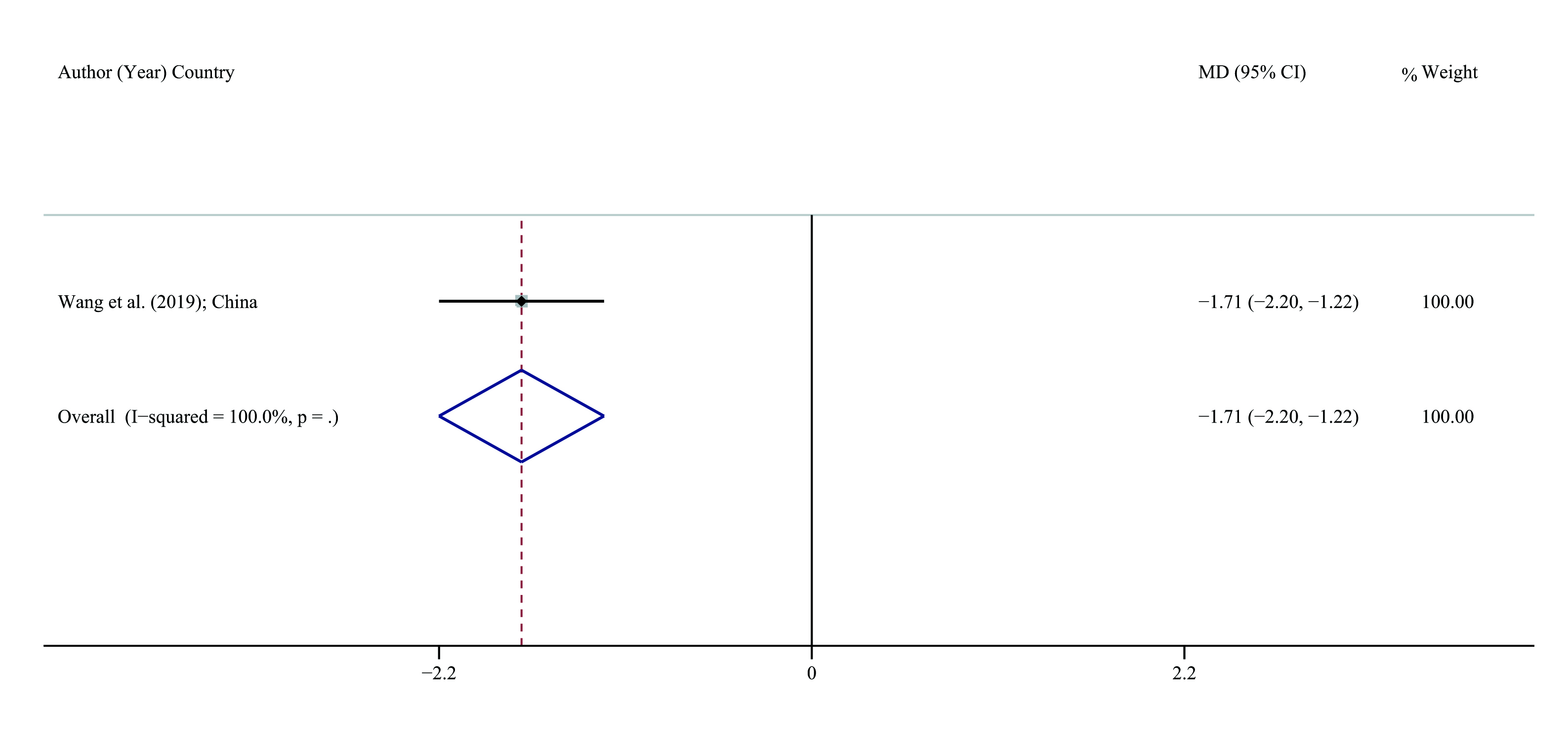


Supplement figure 20: forest plot for mean difference of Fat free mass percentage between GMD and non-GDM group. Each line segment's midpoint shows the mean difference estimate, length of line segment indicates 95% confidence interval (CI) in each study, and diamond mark illustrates the pooled estimate of mean difference.


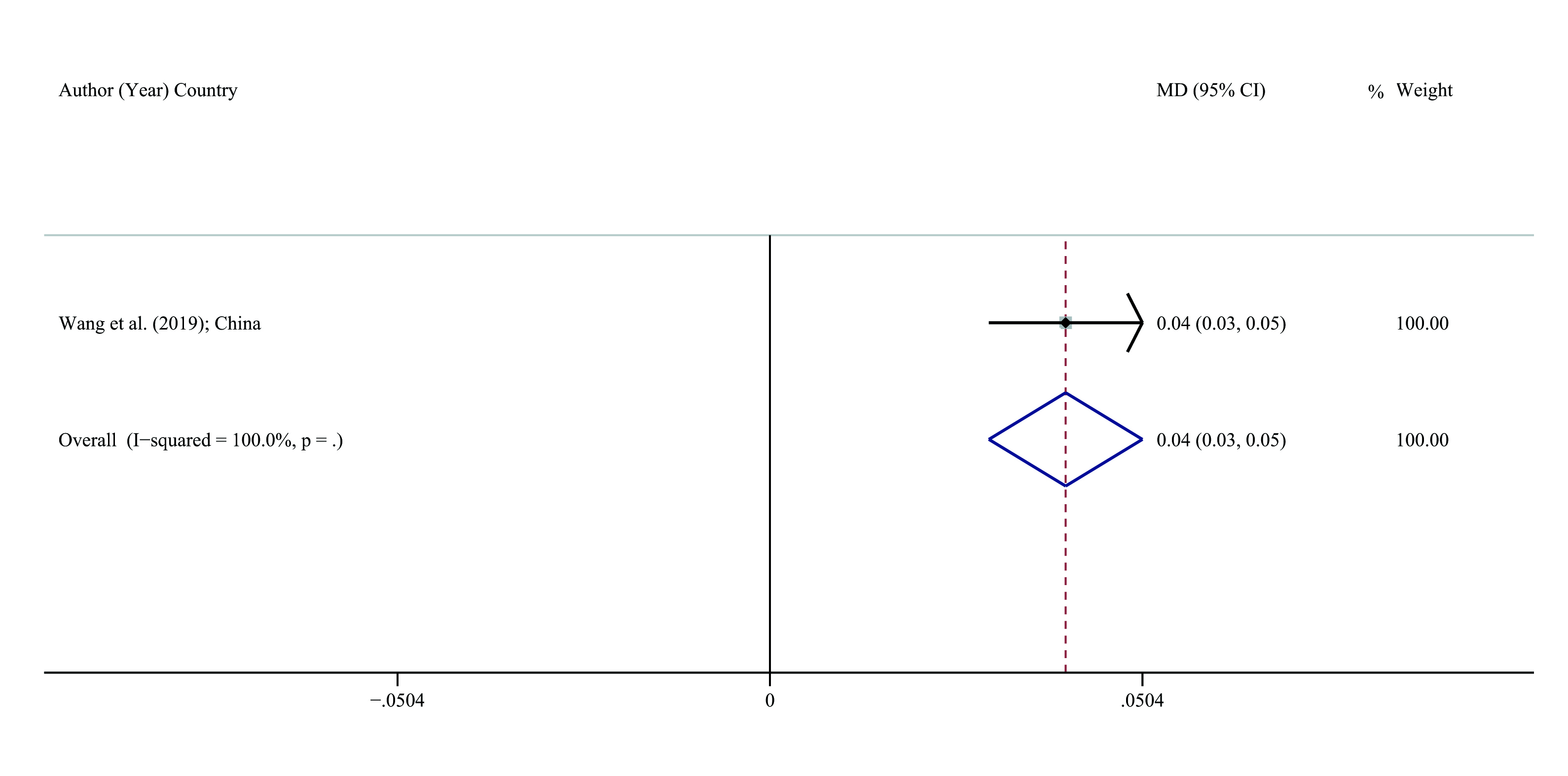


Supplement figure 21: forest plot for mean difference of Fat mass fat free mass ratio between GMD and non-GDM group. Each line segment's midpoint shows the mean difference estimate, length of line segment indicates 95% confidence interval (CI) in each study, and diamond mark illustrates the pooled estimate of mean difference.
